# Understanding the costs and the cost structure of a community-based HIV and gender-based violence (GBV) prevention program: the *Woza Asibonisane* Community Responses Program in South Africa

**DOI:** 10.1186/s12913-020-05385-1

**Published:** 2020-06-10

**Authors:** Bruce Larson, Refiloe Cele, Sarah Girdwood, Lawrence Long, Jacqui Miot

**Affiliations:** 1 Health Economics and Epidemiology Research Office, Department of Internal Medicine,Faculty of Health Sciences, School of Clinical Medicine, University of the Witwatersrand, Johannesburg, South Africa; 2 Department of Global Health, School of Public Health, Boston University, Boston, MA USA

**Keywords:** Costing, Community-based programmes, GBV prevention, HIV prevention

## Abstract

**Background:**

The *Woza Asibonisane* Community Responses (CR) Programme was developed to prevent HIV infections and gender-based violence (GBV) within four provinces in South Africa. The Centre for Communication Impact (CCI) in collaboration with six partner non-governmental organizations (NGOs) implemented the programme, which was comprised of multiple types of group discussion and education activities organized and facilitated by each NGO. To date, little information exists on the cost of implementing such multi-objective, multi-activity, community-based programmes. To address this information gap, we estimated the annual cost of implementing the CR Programme for each NGO.

**Methods:**

We used standard methods to estimate the costs for each NGO, which involved a package of multiple activities targeted to distinct subpopulations in specific locations. The primary sources of information came from the implementing organizations. Costs (US dollars, 2017) are reported for each partner for one implementation year (the U.S. Government fiscal year (10/2016–09/2017). In addition to total costs disaggregated by main input categories, a common metric--cost per participant intervention hour--is used to summarize costs across partners.

**Results:**

Each activity included in the CR program involve organizing and bringing together a group of people from the target population to a location and then completing the curriculum for that activity. Activities were held in community settings (meeting hall, community center, sports grounds, schools, etc.). The annual cost per NGO varied substantially, from $260,302 to $740,413, as did scale based on estimated total participant hours, from 101,703 to 187,792 participant hours. The cost per participant hour varied from $2.8–$4.6, with NGO labor disaggregated into salaries for management and salaries for service delivery (providing the activity curriculum) contributing to the largest share of costs per participant hour.

**Conclusions:**

The cost of implementing any community-based program depends on: (1) what the program implements; (2) the resources used; and (3) unit costs for such resources. Reporting on costs alone, however, does not provide enough information to evaluate if the costs are ‘too high’ or ‘too low’ without a clearer understanding of the benefits produced by the program, and if the benefits would change if resources (and therefore costs) were changed.

## Background

South Africa has one of the highest HIV prevalence rates (18.8%) in the world [1]. To curb transmission of the virus, the South African government has adopted and scaled-up medical interventions such as Post-Exposure Prophylaxis (PEP), Pre-Exposure Prophylaxis (PrEP), Test & Treat (T&T), Voluntary Medical Male Circumcision (VMMC) and condom use [2–4].

To avoid the attenuation of biomedical interventions, both structural and behavioral drivers of HIV also need to be addressed [5, 6]. In South Africa, a number of gender norms and behavioral activities targeting both men and women have been implemented with the aim of reducing risky sexual behavior and intimate partner violence (IPV) and promotion of safe sex, all of which are associated with lowering the risk of HIV infection. Examples of these activities include Stepping Stones [7, 8], Zazi Know Your Strength [9], One Man Can [10], Brothers for Life [11], SKILLZ Zithande [12]. The common theme of these activities is that they involve participatory, knowledge-building seminars/sessions/workshops provided by trained facilitators using a curriculum specifically designed for each activity. While very limited information exists on the effectiveness of these individual activities (e.g., see [13, 14], no information exists on effectiveness when these distinct activities are combined into a package of several of the above activities targeted to different subpopulations in the same location, although efforts are underway [7].

In addition, there is a dearth of studies that report on the costs associated with delivering such activities [15]. For public decision making, understanding costs of multi-activity programs targeting HIV- and GBV-risk reduction is as important as understanding effectiveness (e.g., for economic evaluations using cost-effectiveness analysis). Cost information is also required for developing investment cases to continue to expand scale of implementation. Understanding implementation costs is also needed for budgetary impact and planning purposes, both governments and development funding donors [15].

The Centre for Communication Impact (CCI) worked with NGOs in different locations in South Africa to implement the *Woza Asibonisane* Community Responses (CR) Programme from 2014 to 2019, with funding from United States Agency for International Development (USAID). Each NGO provided an overall package of activities targeted at in-school youth (ages 15–19), out-of-school youth (ages 20–24) and adults (25–49 typically). The overall objectives of the CR Programme were to prevent HIV infections and prevent gender-based violence (GBV) in targeted locations within four provinces (Gauteng, Mpumalanga, Western Cape, KwaZulu-Natal) in South Africa. The primary objective of this analysis was to estimate the cost for each NGO providing the CR package of activities.

## Methods

The cost of implementing any program depends on: (a) who is implementing the package of activities and where; (b) the details of the package; and (c) the additional assumptions and information used to establish costs. We briefly discuss each below.

### Implementing organizations

The CR program was funded by USAID. CCI was the grant ‘prime recipient’, and then six NGOs implemented the program (package of activities) in specific locations within four provinces of South Africa. The six NGOs (with provincial focus) were:

- Heaven’s Defense Force (HDF) – Gauteng;
- Isizinda Sempilo (ISO) – Gauteng;
- The Valley Trust (TVT) – KwaZulu-Natal
- Drama in AIDS Education (DramAidE) – KwaZulu-Natal;
- Project Support Association Southern Africa (PSASA) – Mpumalanga; and
- Grassroot Soccer (GRS) – Western Cape and Gauteng.

Within each province, the NGO organized implementation activities within specific locations to target populations in informal settlements. For reference, **Table S1 in** Additional file 1 lists the 49 locations (wards) where the NGOs operated during the fourth program year (the US government fiscal year 2017, October 2016 to September 2017). The analysis of costs began in January 2018, so the most recent complete year of costs available as of January 2018 was used (using the US government fiscal year because that was also the program year). Additional detail is provided below under costing methods.

### Overview of CR Programme activities

Each NGO implemented a package of activities targeted to HIV and GBV prevention in specific populations. Table 1 provides a list of the target populations and specific activities that comprise the overall package. Individual components of the package targeted the general community, in-school youth (ISY) ages 15–19 years, out-of-school youth (OSY) ages 20–24, adults aged 25 or older, adults who are parents or caregivers, and adult men. Activities for ISY were held in schools, while the other activities were held in other community settings (meeting hall, community center, sports grounds, tents, etc.). These activities can generally be classified as demand-side prevention interventions [16] that bring together a group of individuals in a target population for a facilitated group discussion and completion of an activity-specific curriculum. The goal of these information, education, and communication (IEC) activities were to change knowledge, attitudes, and practices to prevent HIV transmission and GBV.

**Table 1:** Overview of Community Responses Package of Activities

Target population	Activity name	Gender focus	Activity curriculum name (or standard operating procedure)	Intensity of Activity	Participant hours per activity	Number of targeted participants per activity
**The general community and stakeholders**	Community Dialogues	All	Community Responses Dialogue Guide (includes information for all types of dialogues)	1 Workshop = 1 session, 2 h	2	100
**In-school Youth (ISY)** **(15–19 years)**	ISY Dialogues	All	Community Responses Dialogue Guide (includes information for all types of dialogues)	1 Workshop = 1 session, 2 h	2	50
	ISY Gender Norms	All	The SKILLZ Coach’s Guide (July 2016)	1 Workshop = 10 sessions, 1 h each	10	40
	ISY PP Prev	All	Facilitation Plan for HIV Prevention Sessions [Priority Population Prevention (PP_Prev)]	1 Workshop = 1 session, 2 h	2	40
**Out-of-school Youth (OSY)** **(20–24 years)**	OSY Dialogues	All	Community Responses Dialogue Guide (includes information for all types of dialogues)	1 Workshop = 1 session, 2 h	2	100
	OSY Gender Norms	All	Stepping Stones: A training manual for sexual and reproductive health communication and relationship skills	1 Workshop = 10 sessions, 3 h each	30	40
	OSY PP Prev	All	Facilitation Plan for HIV Prevention Sessions [Priority Population Prevention (PP_Prev)]	1 Workshop = 1 session, 2 h	2	40
**Adults (25+)**	Adults Dialogues	All	Community Responses Dialogue Guide (includes information for all types of dialogues)	1 Workshop = 1 session, 2 h	2	100
	Adults Gender Norms	All	Stepping Stones: A training manual for sexual and reproductive health communication and relationship skills	1 Workshop = 10 sessions, 3 h each	30	40
	Adults PP Prev	All	Facilitation Plan for HIV Prevention Sessions [Priority Population Prevention (PP_Prev)]	1 Workshop = 1 session, 2 h	2	40
	Adults Parent Workshop	All	Know Yourself/Know Your Children Parent’s Programme, A Toolkit for Facilitators, Draft 1 - March 2013	1 Workshop = 1 session, 2 h	2	40
	GBV Dialogues	All	Community Responses Dialogue Guide (includes information for all types of dialogues)	1 Workshop = 1 session, 2 h	2	100
	GBV Workshop	Men (ages 20+)	One Man Can	1 Workshop = 5 sessions, 2 h each	10	30

In Table 1, we consider an activity as a combination of the activity and the target population. For example, “dialogues” are one general category of activities in Table 1 targeted to five different populations, with the content and structure of the dialogue tailored somewhat to each population. Dialogues served largely as an awareness raising and demand-creation activity designed to increase awareness of and participation in the other activities targeted to the same population. The ‘community’ dialogues also served the role of seeking largely community input into the overall set of activities and to raise awareness among local leaders, and gain their support, for example, for the other community- and school-based programs.

Each activity listed in Table 1 basically involves organizing and bringing together a group of people (from the target population) in a location and then completing the activity curriculum (in one or multiple sessions depending on the activity). Conceptually, each NGO operated like a company that provides training/education short courses. Locations (venues) and participants need to be identified. Trainers need to be provided (called facilitators), using existing curriculum (see Table 1), and additional materials are perhaps provided either to the facilitators or the participants (typically refreshments). Unlike training companies, the NGOs charged no fees for participation. Conceptually, the refreshments provided some incentive to participate (perhaps more important for the multi-session interventions). Given the variability of the terms “workshops” or “session” in CR programme materials, we have denoted in Table 1 the general ‘intensity’ of the activity based on the number of workshops/sessions, duration in hours, and number of participants.

When disaggregated by target population and activity, each NGO implemented a package comprised of 13 activities. These 13 activities are considered a package (or combination intervention) because all could in principle jointly contribute to HIV and GBV prevention in the program locations and potentially elsewhere. For example, positive externalities could exist if, for example, a young man completing the One Man Can workshop in one location then influenced friends in other locations. For each activity in the package targeting specific subpopulations (Table 1), multiple groups received the same activities (e.g., multiple groups of ISY received the SKILLZ program and HIV prevention sessions) in each location. The numbers of groups receiving these activities varied across the NGOs implementing the program.

To provide the package of activities, each NGO used typical resources for basic program management, oversight, and monitoring and evaluation. While the NGOs implemented these activities, CCI was responsible to USAID for overall program implementation, including program management, monitoring and evaluation, identifying and training for partners, quality assessment and improvement.

### Costing methods

We used standard methods to estimate costs for each NGO implementing the package of activities from the provider’s perspective. These methods have been well-documented elsewhere [17–19]. We briefly describe here how we applied these methods.

From Table 1, each NGO provided activities in specific wards (6–10 wards depending on the NGO); a ward is the smallest administrative geographic boundary. This analysis focuses on the program year from October 2016 to September 2017, which is a budget period that covers the U.S. governments 2017 fiscal year (USG FY 17, or simply FY17 throughout this report). This was the 4th year of the CR program, which reflects a program in ‘full’ or ‘routine’ implementation mode. Start-up costs incurred at the beginning of the program are not included in the analysis, as well as potential trainings provided by CCI (or others) to partners to implement the package of activities. However, equipment purchased in earlier program years is included in the analysis (discussed below).

The ‘cost’ of implementing a program (in this case, the activity package summarized in Table 1) depends on the perspective used in the analysis [17, 18]. This perspective affects which resources (quantities of inputs) are included in the analysis and the unit cost associated with each resource. For the primary perspective, costs from the NGO’s perspective are estimated.

Since the U.S. government, via the United States Agency for International Development, is the funder of the program, costs from this provider’s perspective (combining the partner’s costs with costs of the prime organization – CCI) are also briefly discussed.

Throughout the analysis, key resources used by each NGO are identified even if the NGO incurred no direct cost for the resource. The two main categories of resources used, for which no financial cost was incurred, were: [1] venues for some activities; and [2] time required by the program participants. We stop short of imputing some additional value to these free inputs (which would move the analysis closer to what is often called an ‘social perspective’ or an ‘economic analysis’).

We used a 5% real discount rate to annualize capital purchased (e.g., equipment, vehicles). Based on the purchase price in the year of purchase, an annual equivalent cost based on a 5% real discount rate and an expected useful equipment life (5 years). This annual equivalent cost (AEC) is essentially an implicit annual fee to the program for using the equipment for 1 year. For equipment purchased before FY17, costs were inflation adjusted ‘up’ to 2017. For most NGOs, there were relatively few equipment items (mainly vehicles and computers) for which upfront costs needed to be annualized. Thus, the results are not sensitive to the choice of the discount rate or the useful life of equipment.

All costs and results are reported in 2017 South African Rand (2017 ZAR) and U.S. dollars (USD). As mentioned above, equipment costs incurred before the implementation year (USG FY 17) were adjusted up to the implementation year using the annual consumer price index for South Africa as reported in the International Monetary Fund’s World Economic Outlook database [20]. Summary results were converted to USD 2017 using the annual average exchange rate (13.33 ZAR/USD), which was the annual average for 2017 also reported in the IMF International Financial Statistics [20]. In addition, the 13.33 ZAR/USD rate is consistent with the actual rate used by USAID to convert transfers to CCI from Dollars to Rand during October 2016 to September 2017 (the costing year).

Different strategies or approaches can be used for evaluating costs based on the information available and purpose of the analysis. Terms such as a bottom-up approach, or a top-down approach, or an ingredients-based approach, or an expenditure-based approach are sometimes used to categorize the type of analysis used. A combination of these approaches was used in this analysis, but in general a combined expenditure- and ingredients-based approach was used [21]. An ingredients-based approach requires information on the quantity of each type of input and a price (unit cost) for each, and then total costs for this input (or inputs included in the ingredients analysis) are simply the price multiplied by the quantity. However, for other inputs (e.g. labour) and/or activities (monitoring and evaluation), a more aggregated expenditure-based approach is used [21]. In Additional file 2**,** a detailed example for one NGO (DramAidE) is provided.

## Results

Table 2 summarizes the overall implementation activities for each NGO based on the number of each activity listed in Table 1. Based on participant hours per activity listed in Table 1, Table 2 summarizes total participant hours by activity for each NGO. Total costs (USD) for each NGO and cost per participant hour (USD) are also included in Table 2.

**Table 2:** Total number of activities, participant hours, and costs by NGO

Total number of activities by NGO						
Activity Name	DramAidE	ISO	TVT	PSASA	HDF	GRS
Community Dialogues	16	16	20	12	18	16
ISY Dialogues	16	8	20	27	9.9	16
ISY Gender Norms	16.8	16	18	27	4.5	28
ISY PP Prev	62.4	96.8	28	234	45.9	139.2
OSY Dialogues	8	8	10	6	9	8
OSY Gender Norms	20	16.8	20	24.6	9	28
OSY PP Prev	66.4	141.6	45	227.4	68.4	188
Adults Dialogues	8	8	10	6	9	8
Adults Gender Norms	56	31.2	50	25.8	36	63.2
Adults PP Prev	183.2	312.8	108	306	196.2	214.4
Adults Parent Workshop	30.4	31.2	12	22.2	19.8	56.8
GBV Dialogues	16	32	10	24	36	32
GBV Workshop	10.4	10.4	13	9.6	4.5	16.8
**Total Participant Hours by NGO**						
	**DramAidE**	**ISO**	**TVT**	**PSASA**	**HDF**	**GRS**
Community Dialogues	3200	4800	4000	2400	3600	3200
ISY Dialogues	1600	1200	2000	2717	964	1600
ISY Gender Norms	6800	12,800	14,400	32,612	5786	11,200
ISY PP Prev	4990	7760	2243	18,729	3668	11,144
OSY Dialogues	1600	2400	2000	1200	1800	1600
OSY Gender Norms	24,000	13,600	16,000	29,640	10,800	33,600
OSY PP Prev	5288	11,346	3600	18,178	5468	15,068
Adults Dialogues	1600	2400	2000	1200	1800	1600
Adults Gender Norms	67,200	12,400	40,000	30,927	43,200	75,600
Adults PP Prev	14,644	25,054	8600	24,462	15,680	17,120
Adults Parent Workshop	2400	2480	960	1779	1609	4560
GBV Dialogues	3200	9600	2000	4800	7200	6400
GBV Workshop	4000	1500	3900	2835	1800	5100
**Total Participant Hours**	**140,522**	**107,340**	**101,703**	**171,479**	**103,374**	**187,792**
Costs (USD)						
Total Cost (USD	599,952	260,302	376,131	441,088	294,628	740,413
Cost per participant hour (USD)	4.27	2.43	3.70	2.57	2.85	3.94

While each NGO implemented the overall package of activities as outlined in Table 1, NGO’s organized different numbers of each specific activity, in part because they operated in more locations, but also in part because NGOs organized relatively different numbers of activities (in each location/ward). For example, for DramAidE, participant hours for Adult Gender Norms workshops were substantially larger than participant hours for OSY Gender Norms workshop. For ISO, however, OSY Gender Norm hours were larger than Adult Gender Norm hours.

Total costs ranged from a low of about R3.5 million ($260,302) to R9.9 million ($740,413). While total costs are provided in Table 2, cost profiles for each NGO, with costs disaggregated by key input or activity categories, are presented in Additional file 3. An example of a cost profile is provided in Table 3 (for DramAidE, the NGO used as the example in Additional file 2).

**Table 3:** Summary cost profile for DramAidE

DramAidE			
Headquarters	Rand 2017	Rand 2017	Share of total participant hours
Salary (Management)	3,621,944	3,621,944	
Salary (Service delivery)	1779,840	1779,840	
Office	707,140	1,472,175^c^	
Office (M&E licenses)	2508		
Equipment (AEC)	38,078		
Vehicle (AEC)	57,414		
Travel	474,341		
Prior Equipment (AEC)	27,467		
Prior Vehicle (AEC)	114,827		
Cell phone top up for staff^b^	50,400		
**Direct**^a^			
Community Dialogues	43,000	43,000	0.023
ISY Dialogues	12,000	12,000	0.011
ISY Gender Norms	102,000	102,000	0.048
ISY PP Prev	18,712	18,712	0.036
OSY Dialogues	21,500	21,500	0.011
OSY Gender Norms	160,000	160,000	0.171
OSY PP Prev	39,660	39,660	0.038
Adults Dialogues	21,500	21,500	0.011
Adults Gender Norms	448,000	448,000	0.478
Adults PP Prev	109,831	109,831	0.104
Adults Parent Workshops	36,000	36,000	0.017
GBV Dialogues	42,200	42,200	0.023
GBV Workshops	60,000	60,000	0.028
**Total**	**7,988,362**	**7,988,362**	

^a^Direct costs were refreshments for participants and minor venue costs for some non-school locations

^b^Cell phone top up time for staff to link PP Prev participants to HIV testing locations

^c^All office, headquarters costs combined into one aggregate ‘office’ category

In Table 3, the three main cost categories were for staff (management), staff (service delivery), and then overall “office” costs (e.g., rent, utilities, equipment, travel for staff, etc.). The management staff details for DramAidE are include in Additional file 2, but include for example the Director, the Project Manager, financial staff, project officers, and other headquarters staff. The service delivery staff are called community mobilizers or facilitators, who worked in specific locations to lead the activities listed in Table 1.

In Table 3, office costs included numerous items but the largest were rent, an audit, and utilities. Regarding equipment, the NGO purchased new equipment, which was then converted to an annual equivalent cost, and used equipment obtained in prior years. Travel was mainly for headquarters staff to CCI headquarters for quarterly meetings along with travel reimbursement for staff using their own vehicle. In Table 3, the items from Office to Cell phone top up are aggregated into one overall “office” cost category.

In Table 3, the “direct” costs for each activity are simply any costs specifically linked to each activity in the NGOs supporting materials (budgets, scopes of work). Essentially, for each of the activities, participants received some refreshments (at each session). Additional costs were incurred for the venue location for community dialogues and the Stepping Stones (Gender Norms) activities for out-of-school youth and adults. Nonetheless, refreshments remained the majority of direct costs for these activities with (minor) venue costs.

**Fig. 1:**
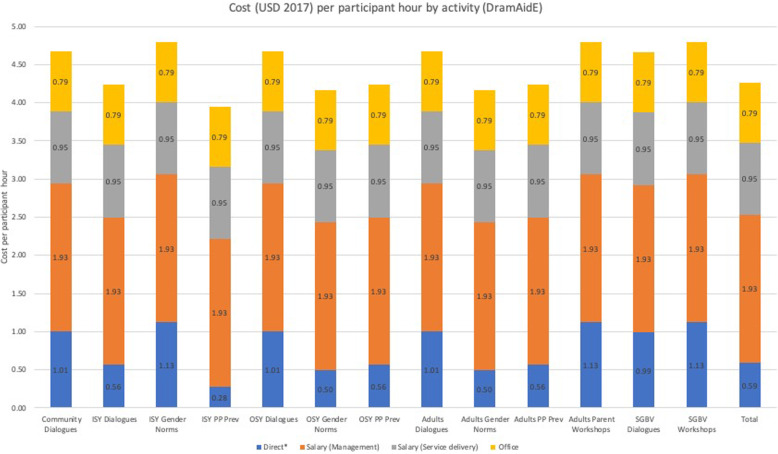
Cost per participant hour (USD) by activity disaggregated by cost category (DramAidE)

Table 3 also includes the share of total participant hours for each of the specific activities (based on the hours provided in Table 2). If the 3 major cost categories in Table 3, salaries (management), salaries (service delivery), and the aggregated office costs are apportioned to each activity based on participant hour share, the cost per participant hour reported in Table 2 can then be disaggregated into the 3 major cost categories and direct costs for each activity and for the total NGO program. Figure 1 summarizes this information for DramAidE.

In Fig. 1, costs per participant hour of $4.26 reported in Table 2 can be separated into direct costs ($0.59), management salary ($1.93), salary for service delivery ($0.95), and aggregated office costs ($0.79). Little variation exists in cost per participant hour across activities because refreshments comprised the majority of such costs, and refreshment costs per person per session were similar across DramAidE’s activities (but vary per hour because hours per session varied). A Fig. 1 for each of the six NGOs is provided in Additional file 3.

**Fig. 2:**
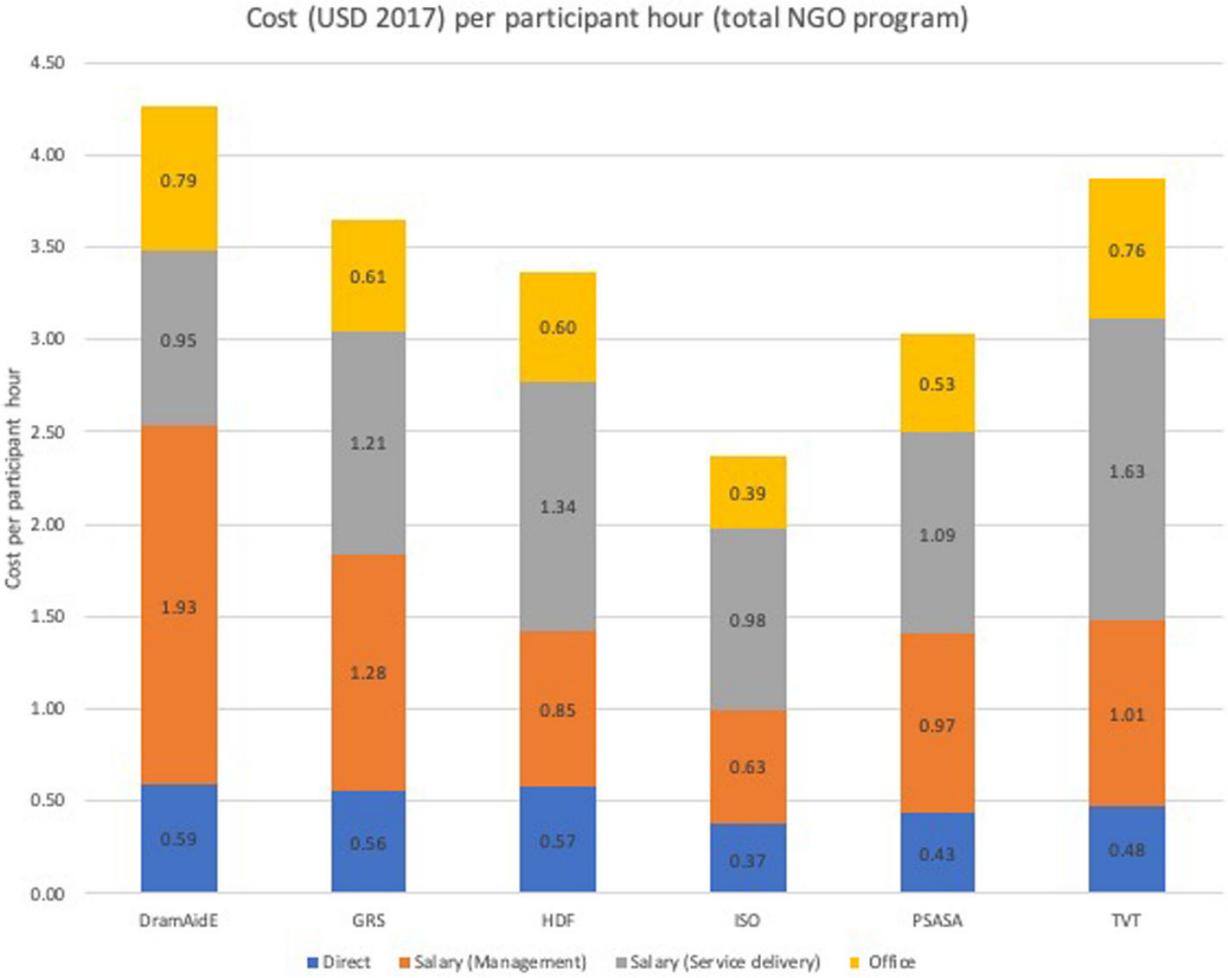
Cost per participant hour (USD) for each NGO disaggregated by cost categories

Figure 2 summarizes the cost per participant hour (USD) for each NGO disaggregated by the four cost categories included in Fig. 1.

For each NGO, labor costs, either salary for management or salary for service delivery (the facilitators of the activities), were the major cost categories. The direct costs per participant hour for each activity, mainly refreshments, where the smallest share for all NGOs, followed by the aggregated headquarters-based costs.

Based on cost per participant hour and total participant hours from Table 2, no obvious economies of scale existed across the six NGOs. Cost per participant hour varied substantially for the three NGOs with similar scale (101,703, 103,374, and 107,349 participant hours), while the remaining three varied on scale and cost per participant hour, but with no obvious pattern.

Some possible differences across provinces are observed in Fig. 2. DramAidE and TVT both operated in KwaZulu Natal Province, which had the largest and similar estimated costs per participant hour, but with some differences in costs of labor for management and service delivery. ISO and TVT both operated in Gauteng Province, with differences in costs per participant hour. GRS operated in both Gauteng and Western Cape, but with similar costs per participant hour as with HDF.

## Discussion

The *Woza Asibonisane* Community Responses (CR) Programme was implemented by six NGOs, working in separate locations in South Africa, to provide a package of activities targeted to multiple groups, with goals of reduction HIV and GBV risks. The overall structure of activities was similar for in-school youth (ages 15–19), out-of-school youth (ages 20–24), and adults (ages 25+): dialogues; short HIV prevention workshops (2 h); and more intensive workshops address gender-based violence (10-h workshops using SKILLZ or Stepping Stones curricula). To implement these activities, the NGOs needed to identify participants and locations (venues) for the activities. The NGO provided facilitators (trainers/educators) to implement the curriculum. Additional materials were provided to the facilitators (e.g., curriculum materials) and participants (refreshments). The cost per participant hour varied from $2.36–$4.26.

Not surprisingly, for a program that largely uses staff to organize and provide activities, salary costs comprised the largest component of program costs for all partners. This is also the case in other community-based programs, such as the SASA! program in Uganda, where staff-related costs were 76% of total program costs (salaries and training) [22]. Within the category of CR program staff costs, costs for general program “management” (excluding salaries for facilitators and community mobilizers) were the largest cost category for two NGOs while salary for service delivery (the facilitators and community mobilizers) were the largest cost category for the other four. The term “management” is used here to encompass all the supporting activities needed to implement the CR intervention package, which include: financial management and reporting; other grants management and reporting activities; staff hiring and oversight and training; monitoring and evaluation activities. As noted in the previous paragraph, NGOs needed to identify and enroll participants for the various activities. Information is not available to document costs specifically related to this issue, but such activities were completed in part by both service delivery and management, so such costs are included within the respective cost categories.

A costing analysis cannot assess whether such costs are too large or too small without additional information on costs and effectiveness of multiple programs (e.g., reducing management costs could lead to worse outcomes).

To contextualize the costs calculated in our analysis, a recent review of the literature on the costs of HIV services in South Africa provides some useful information for understanding the costs of other types of HIV services [23]. For example, for the two mass media campaign costs included in the literature review, the cost per person “reached” were $0.25 and $82, although no information is provided to explain the large range. Education targeting the use of male and female condoms cost $5 per person, which is roughly similar to an hour of participant time in the CR program.

Unfortunately, little published, peer-reviewed, information exists on the costs of implementing the activities included in the CR program for comparison purposes. The few studies that have been published focus on measuring the impact or benefits of their respective programs. An evaluation study of the Stepping Stones activity was published in 2008 [13]. In this study, the Stepping Stones activity involved 50 h rather than 30 h of participant time (as included in the CR programme), and the authors concluded that the intervention reduced risk factors relating to HIV and intimate partner violence. Unfortunately, no cost information was reported, and possible effectiveness of a 50-h Stepping Stones activity compared to a 30-h version included in the CR program does not exist. An additional study evaluated a program combining Stepping Stones with another intervention (a livelihoods intervention called Creating Futures) [24]. While the protocol manuscript for this study indicates that implementation costs for this combined intervention will be evaluated, results are yet to be reported [7]. A recent systematic review reports on the cost-effectiveness of gender-responsive interventions [25], and some "gender-empowerment, community mobilization" activities were considered cost effective.

The current analysis is not without limitations. First, this analysis focuses on 1 year (October 2016 to September 2017). This was the fourth year of the CR Program, so the cost presented here are interpreted as a ‘typical’ program year. If the type of program was to be rolled out to new locations, additional costs for training staff would likely be needed. In addition, the question of free venues or venues requiring hiring should be assessed from the beginning.

Second, this analysis relied mainly on the program budgets and scopes of work as the source documents for the analysis, combined with other information (expenditures on equipment purchased previously for the program) and discussions with the NGO staff. When budgets are organized with a structure that is not clearly related to program activities, a costing analysis must generally rely on program expenditures as the foundation for the analysis. In case of the CR Program NGO budgets, however, the budgets are presented with categories clearly linked to specific interventions in the program. As long as implementation did not deviate significantly from the scope of work (SOW) and budget, in which case the partner and CCI would have reported such changes to USAID for approval, then the budgets should track expenditures fairly closely.

Thirdly, due to data availability limitations, participation and attrition rates, particularly for interventions that require multiple attendance, are unknown. For example, with cost per participant hour denoted as “c”, attrition denoted as “a” or lack of participation would be expected to increase costs per participant hour to c/(1-a). For example, as a rough estimate, if the program only provided 50% of intended participant hours, cost per participant hour would double. Because most of the NGOs costs are not related to such attrition, any possible savings from attrition (e.g., save a bottle of water for a next session), would be minor.

Finally, cost per participant hour does not provide enough information to evaluate if the costs are ‘too high’ or ‘too low’ compared to other community-based HIV and GBV prevention programs as well as other types of HIV-related services. Also, it worth noting that our costing methodological approach focuses only on financial expenditure employing a mixture of bottom-up and top-down methods, based on program budgets with pre-determined line items which may not account for other indirect costs associated with delivering the program [26].

There are a few possible ways to measure the benefits of the CR Program going forward. First, given that the CR program is designed for prevention of HIV and GBV, a part of the possible contribution of such community-based programs is to increase demand for HIV prevention services (as well as GBV services). Increasing demand for HIV testing, male circumcision, PrEP, early presentation for antenatal care when pregnant, complements these other HIV prevention activities. Measuring and documenting such outcomes is relatively feasible and a potentially important topic for future program M&E activities. Second, with direct behavior change to reduce HIV risks (e.g., safer sex) among the program participants (and their non-participant partners), as well as reduced risks following additional biomedical interventions induced in part by the program, some cases of HIV are likely to be delayed or avoided over a lifetime. This of course is a key question that is unlikely to be directly answered by one or even a few studies. The impacts of this specific program or other similar programs on HIV transmission, are unlikely to be answered due in part to the complexity of the issues.

Most studies to date have focused on the impact of community-based GBV programs, but none to date have looked at the cost of actually implementing these programs. Ideally, once additional information is reported on the multiple potential benefits of community-based programs such as the CR program, the cost and the benefits can be brought together and compared to other HIV and GBV prevention efforts.

## Conclusion

This analysis has produced estimates of the range of costs that can be expected from implementing a package of demand-side prevention activities such as those of the *Woza Asibonisane* Community Responses (CR) Programme. While community-based activities that address social and structural drivers of HIV may prevent new HIV infections and reduce sexual- and gender-based violence, it is however important to understand the costs associated with implementing such programmes for budgetary impact and planning purposes.

## Supplementary information

**Additional file 1.** CR Programme implementation locations.

**Additional file 2.** A detailed example of costing methods.

**Additional file 3.** Summary cost profiles of implementing NGOs.

## Data Availability

The datasets used and/or analysed during the current study are available from the corresponding author on reasonable request.

## Abbreviations

CCI: Centre for communication impact
DramAidE: Drama in AIDS education
GBV: Gender-based violence
GRS: Grassroot soccer
HDF: Heaven’s defense force
IEC: Information, education, and communication
IPV: Intimate partner violence
ISO: Isizinda sempilo
ISY: In-school youth
NGO: Non-governmental organization
OSY: Out-of-school youth
PEP: Post-exposure prophylaxis
PrEP: Pre-exposure prophylaxis
PSASA: Project support association Southern Africa
SOW: Scope of work
T&T: Test & treat
USAID: United States agency for international development
USG FY: U.S. governments fiscal year
VMMC: Voluntary medical male circumcision

## References

[1] World Health Organization. Prevalence of HIV among adults aged 15 to 49 Estimates by country 2018 [Available from: http://apps.who.int/gho/data/view.main.22500?lang=en.

[2] Bekker L-G, Rebe K, Venter F. Southern African guidelines on the safe use of pre-exposure prophylaxis in persons at risk of acquiring HIV-1 infection. S Afr J HIV Med. 2016;17(1):455. 10.4102/sajhivmed.v17i1.455 PMC5843155 29568613

[3] National Department of Health RoSA. National consolidated guidelines for the prevention of mother-to-child transmission of HIV (PMTCT) and the management of HIV in children, adolescents and adults. Pretoria: National Department of Health Pretoria; 2015.

[4] National Department of Health RoSA (2010). National HIV counselling and testing policy guidelines.

[5] Bekker L-G, Beyrer C, Quinn TC (2012). Behavioral and biomedical combination strategies for HIV prevention. Cold Spring Harb Perspect Med.

[6] Parkhurst JO (2014). Structural approaches for prevention of sexually transmitted HIV in general populations: definitions and an operational approach. J Int AIDS Soc.

[7] Gibbs A, Washington L, Willan S, Ntini N, Khumalo T, Mbatha N (2017). The stepping stones and creating futures intervention to prevent intimate partner violence and HIV-risk behaviours in Durban, South Africa: study protocol for a cluster randomized control trial, and baseline characteristics. BMC Public Health.

[8] Welbourn A. Stepping stones. A package for facilitators to help you run workshops within communities on HIV/AIDS communication and relationship skills 1995.

[9] Durden E, Gumede M. Zazi Know Your Strength: A toolkit for facilitators. Available from: https://www.zazi.org.za/sites/default/files/6044_zazi_toolkit.pdf.

[10] Sonke Gender Justice Network. One Man Can Toolkit 2006.

[11] Johns Hopkins Health and Education in South Africa & Sonke Gender Justice. Brothers For Life Facilitators Guide. 2010.

[12] Grassroot Soccer. SKILLZ Zithande curriculum. 2016.

[13] Jewkes R, Nduna M, Levin J, Jama N, Dunkle K, Puren A (2008). Impact of stepping stones on incidence of HIV and HSV-2 and sexual behaviour in rural South Africa: cluster randomised controlled trial. Bmj..

[14] Kaufman Z, DeCelles J, Nkosi Z. GOAL Trial: pilot results of a sport-based HIV prevention intervention to inform a cluster-randomized trial in South African schools. Washington DC: XIX International AIDS Conference; 2012.

[15] United Nations Population Fund. Costing the three transformative results. New York; UNFPA; 2019.

[16] Krishnaratne S, Hensen B, Cordes J, Enstone J, Hargreaves JR (2016). Interventions to strengthen the HIV prevention cascade: a systematic review of reviews. Lancet HIV.

[17] Husereau D, Drummond M, Petrou S, Carswell C, Moher D, Greenberg D (2013). Consolidated health economic evaluation reporting standards (CHEERS) statement. Cost Effectiveness Resour Allocation.

[18] Drummond MF, Sculpher MJ, Claxton K, Stoddart GL, Torrance GW. Methods for the economic evaluation of health care programmes. New York: Oxford university press; 2015.

[19] Larson BA, Wambua N (2011). How to calculate the annual costs of NGO-implemented programmes to support orphans and vulnerable children: a six-step approach. J Int AIDS Soc.

[20] World Economic Outlook Databases [Internet]. 2017 [cited 18 October 2018]. Available from: https://www.imf.org/en/Publications/SPROLLS/world-economic-outlook-databases#sort=%40imfdate%20descending.

[21] Cunnama L, Sinanovic E, Ramma L, Foster N, Berrie L, Stevens W (2016). Using top-down and bottom-up costing approaches in LMICs: the case for using both to assess the incremental costs of new technologies at scale. Health Econ.

[22] Abramsky T, Devries K, Kiss L, Nakuti J, Kyegombe N, Starmann E (2014). Findings from the SASA! Study: a cluster randomized controlled trial to assess the impact of a community mobilization intervention to prevent violence against women and reduce HIV risk in Kampala, Uganda. BMC Med.

[23] Meyer-Rath G, van Rensburg C, Chiu C, Leuner R, Jamieson L, Cohen S (2019). The per-patient costs of HIV services in South Africa: systematic review and application in the south African HIV investment case. PLoS One.

[24] Jewkes R, Gibbs A, Jama-Shai N, Willan S, Misselhorn A, Mushinga M (2014). Stepping stones and creating futures intervention: shortened interrupted time series evaluation of a behavioural and structural health promotion and violence prevention intervention for young people in informal settlements in Durban, South Africa. BMC Public Health.

[25] Remme M, Siapka M, Vassall A, Heise L, Jacobi J, Ahumada C (2014). The cost and cost-effectiveness of gender-responsive interventions for HIV: a systematic review. J Int AIDS Soc.

[26] Remme M, Michaels-Igbokwe C, Watts C. What works to prevent violence against women and girls? Evidence review of approaches to scale up VAWG programming and assess intervention cost-effectiveness and value for money. 2014.

