# Supplementary material for: Understanding the costs and the cost structure of a community-based HIV and gender-based violence (GBV) prevention program: the Woza Asibonisane Community Responses Program in South Africa
Source: BMC Health Serv Res. 2020 Jun 10;20:526. doi: 10.1186/s12913-020-05385-1 (PMC7288692; doi:10.1186/s12913-020-05385-1)
Supplement: Supplementary file 1 — Additional file 1. CR Programme implementation locations. [file 12913_2020_5385_MOESM1_ESM.pdf]

Table S1. NGO Implementation Locations

Partner	Province	District or Metropolitan Municipality	Local Municipality (or Sub District)	Ward Number
DramAidE	KZN	EThekwini	EThekwini Central	25
	KZN	EThekwini	EThekwini Central	88
	KZN	EThekwini	EThekwini Central	101
	KZN	EThekwini	EThekwini South	99
	KZN	Ugu	Hibiscus Coast	3
	KZN	Ugu	Hibiscus Coast	20
	KZN	UThungulu	UMhlathuze	1
	KZN	UThungulu	UMhlathuze	7
GRS	GP	Johannesburg	Region E	75
	GP	Johannesburg	Region G	6
	GP	Johannesburg	Region G	8
	WC	Cape Town	Mitchells Plain	33
	WC	Cape Town	Southern subdistrict	80
	WC	Cape Town	Western subdistrict	104
	WC	Cape Town	Klipfontein	40
	WC	Cape Town	Khayelitsha	95
HDF	GP	Tshwane	Region 1	24
	GP	Tshwane	Region 1	25
	GP	Tshwane	Region 1	26
	GP	Tshwane	Region 1	90
	GP	Tshwane	Region 3	71
	GP	Tshwane	Region 3	72
	GP	Tshwane	Region 6	10
	GP	Tshwane	Region 6	17
	GP	Tshwane	Region 6	40
ISO	GP	Johannesburg	Region A	95
	GP	Johannesburg	Region A	96
	GP	Johannesburg	Region A	113
	GP	Johannesburg	Region D	19
	GP	Johannesburg	Region D	30
	GP	Johannesburg	Region D	24
	GP	Sedibeng	Emfuleni	23
	GP	Sedibeng	Emfuleni	36
PSASA	MP	Gert Sibande	Govan Mbeki	12
	MP	Gert Sibande	Govan Mbeki	13
	MP	Gert Sibande	Govan Mbeki	14
	MP	Gert Sibande	Govan Mbeki	19
	MP	Ehlanzeni	Mbombela	14

	MP	Nkangala	Emalahleni	12
TVT	KZN	Ethekwini	North Central	34
	KZN	Ethekwini	North Central	55
	KZN	Ethekwini	North Central	60
	KZN	Ethekwini	North Central	61
	KZN	Ethekwini	North	39
	KZN	Ethekwini	North	53
	KZN	Ethekwini	North	56
	KZN	Ethekwini	West	19
	KZN	Ethekwini	West	23
	KZN	Ethekwini	West	92

3
