# Supplementary material for: Understanding the costs and the cost structure of a community-based HIV and gender-based violence (GBV) prevention program: the Woza Asibonisane Community Responses Program in South Africa
Source: BMC Health Serv Res. 2020 Jun 10;20:526. doi: 10.1186/s12913-020-05385-1 (PMC7288692; doi:10.1186/s12913-020-05385-1)
Supplement: Supplementary file 2 — Additional file 2. A detailed example of costing methods. [file 12913_2020_5385_MOESM2_ESM.pdf]

A detailed example -- Drama in AIDS Education (DramAidE)

We generally followed the process for estimating the annual costs of NGO programs published previously (see [4]). We first worked with CCI and the partners to obtain the partner's Scope of Work (SOW) and budget for the costing year (USG FY17). The SOW provided the neccessary detailed information on implementation locations, types and number of interventions for each target population, etc. The budgets provided the detailed, line item information inputs and prices (unit costs). Additional information and clarification was then obtained through follow up discussions with CCI and partners.

In the remainder of this section (IV), we walk through the detailed process followed for one partner (DramAidE). This same process was followed for all six partners. The detailed analysis for each partner is contained in one Excel Workbook file (provided as an electronic annex to this document). Reviewing the DramAidE example in detail will also make it easier to review the analyses for the other partners because the process was identical and the Workbook for each partner is organized in the same fashion. We recommend that this section is read while also reviewing the DramAidE Workbook.

The first sheet in the Workbook (titled A – Overview) is essentially a table of contents with hyperlinks to the main worksheets (labeled B-F).

In the sections below, we walk through the process used to convert the information in the budget (sheet F) and SOW to create a detailed cost profile (sheet E). The detailed cost profile is then aggregated into a summary cost profile (sheet C). The summary cost profile (sheet C) is then combined with program information (sheet B) to present costs and 'outputs' together (sheet D). The term 'output' will be described in more detail below.

To maintain confidentiality of salary information, the actual budget sheet (sheet F) is hidden and password protected). Other than the individual salary information, all the other information in the budget is provided in additional worksheets as explained below.

The overall DramAidE budget for FY 17 was R8.2 million ($0.61 million), and the program operated in 8 wards. The budget was organized into 12 major categories. The processes used for converting each category in the budget into companion information in the detailed cost profile is presented below.

Category 1. Personnel

Budget category 1 included salaries, fringe benefits, and tax payments for the staff members listed in Table 3 below. These staff members were primarily responsible for program management and administration or service delivery (e.g., organizing and providing the interventions to the target populations).

Table 3. Staff Categories and levels of effort (LOE) from Budget

**Budget description**	**LINE ITEM DESCRIPTION**	**Project Role**	People	LOE (as proportion of 1 full-time person)
Salary	Director	Management	1	0.95
Salary	Project Manager	Management	1	0.95
Salary	Finance Manager	Management	1	0.98
Salary	Finance and admin officer	Management	1	0.98
Salary	Quality Improvement Manager	Management	1	1
Salary	Project officers	Management	4	1
Salary	M & E Officer	Management	1	1
Salary	HTS Coordinator	Management	1	1
Salary	Admin/general office assistant	Management	1	1
Salary	Community facilitators	Service Delivery	8	1
Salary	Community Mobilizers	Service Delivery	16	1
Salary	Community Mobilizers	Service Delivery	2	0.75
Salary	Community Facilitators	Service Delivery	2	0.75

Salary costs by location and project role were then aggregated (see sheet 1. Personnel in the workbook and provided in the table below). This is an example where an 'expenditure-based' approach is used.

In addition, there is a budget category 2 that included R14,460 for staff capacity building (mainly providing the SKILLZ intervention and the Parent's Workshops). A separate sheet was not created for this small amount, and the amount was simply included in the total staff category (as service delivery in sheet 1. Personnel). The assumption here is that this training was useful for the current program year.

From Sheet 1. Summary Personnel Costs by Project Role

**Project Role**	**Total with extra tax payments**
Management	3,621,944
Service Delivery	1,794,300
Total	5,416,244

This information is included as the first line items in the detailed cost profile in the workbook (see the sheet E. Detailed Cost Profile, rows 2-3).

Category 3. Community Dialogues

Sheets numbered 3-8 in the DramAidE workbook walk through additional costs for each intervention provided by the program. The community dialogues (see Table 2 intervention package) are targeted to the general community. These dialogues are used in part to introduce the CR programme to the local community (at the beginning of a year), which facilitates participation by community members in other CR program interventions. The information from this budget category was first copied into a separate workbook sheet (sheet 3. Comm Dialogues). A copy of the budget information is provided below. Besides the staff who helped organize and facilitate these dialogues, minor additional costs were allocated to refreshments and the venue hire.

From sheet 3. Community dialogues as budgeted

**3. COMMUNITY DIALOGUES**						
**Conduct Community Dialogues 2 x Community Dialogues in 8 x wards targeting 100ppl per ward) (Activity 2.2)**	**Days**	**# of Wards**	**Units**	**Unit Description**	**Rate**	**Total**
Refreshments @R 15pp x100 people per dialogue x 8 wards	2	8	100	participants	R 15.00	R 24,000.00
Venue per day @R250 per day x 8 wards	2	8	1	venue	R 250.00	R 4,000.00
Additional tents hire & chairs for 5 sites (Ward 7, 25, 99, 88, 20) which do not have venues.	2	5	1	Venue	R1,500.00	R 15,000.00
**TOTAL COMMUNITY DIALOGUES**						**R 43,000.00**

Based on the above information, a basic community dialogue involved a cost of R1,750 (R15 per person for 100 participants and R250 for a basic venue fee). If a tent was also needed (for the 5 wards noted), then the dialogue cost was R3,250.

Based on the ward numbers with tents (3 in EThekwini District, 1 each in the other two districts), a weighted average cost of a dialogue per ward computed (see below).

From sheet 3. Community dialogue cost

District	Location Number	Dialogues per ward	Wards with a tent	Wards no tent	Cost per dialogue (weighed average)

EThekwini	2	2	3	1	R2,875
Ugu	3	2	1	1	R2,500
UThungulu	4	2	1	1	R2,500

Community dialogues require the participation of various community members (see the SOW for detail). If these 2 dialogues last 2 hours each, and 100 community members participate, the community is essentially contributing 400 hours per ward and 32,000 hours across the 8 wards. This is roughly the equivalent of 15 full-time levels of effort (8 hours per day, 260 working days be per year).

For the program to operate, this is a key input category (community time contributed to dialogues) that is not explicitly included in program costs.

The basic information for community dialogues—number per ward; cost per dialogue (from sheet 3. Comm Dialogues)—is then included in the detailed cost profile (sheet E, rows 4-6).

Category 4.1. In-school Youth (ages 15-19)

After community dialogues, budget category 4.1 was for interventions targeted at in-school youth (ISY) ages 15 to 19. As noted in Table 2, three sets of activities (interventions) were targeted at in-school youth: (1) dialogues with the school community (ISY dialogues, 2 hours); (2) the SKILLZ intervention (noted as Gender Norms for USAID purposes; 1 workshop = 10 sessions, 3 hours each); and (3) an HIV prevention intervention (called PP Prev –priority population prevention; 1 workshop = 1 session, 2 hours).

Budget category 4.1 includes the first two (dialogues and SKILLZ). Information for the third intervention, the HIV prevention intervention (the 1 session over 2 hours using the PP_Prev facilitators guide), was included in Budget category 6.

The information for budget category 4.1 was copied into a new sheet (4.1. ISY). For ISY dialogues, the budget information is:

From sheet 4.1 ISY – School dialogues as budgeted

**4.1 IN-SCHOOL PROGRAMME 15-19 YR. OLDS (Activity 2.3 number 1.)**	**Dialogues**	**# of schools**	**Units**	**Unit Description**	**Rate**	**Total**
Refreshments @ R15.00 pp x 50 people x 8 schools x 2 dialogues	2	8	50	participants	15	R12,000

As with community dialogues, this information was reorganized into a simple cost per dialogue (50 times R15 = R750), with 2 dialogues per ward, for a total of R1,500 per ward. This information is then included in the cost profile (budget category 4.1, ISY dialogues).

The next intervention targeted to ISY was the SKILLZ curriculum (workshop), implemented with a group of students (40) over 10 separate sessions, each lasting approximately 1 hours (10 hours total per student). The information from the budget is provided below.

From sheet 4.1 ISY – SKILLZ workshops as budgeted

Delivery of SKILLZ Curricula with 15 - 19yr .olds	Classes	Sessions	Units	Unit Description	Rate	Total
Refreshments	17	10	40	participants	15	R10,2000

With 17 total classes (workshops) in the budget, on average 2.125 classes per ward (typically in the same school) received the SKILLZ curriculum. The cost per workshop (refreshments) was R6,000 (10 times 40 times R15).

From sheet 4.1 ISY SKILLZ Workshop cost per ward

ISY SKILLZ Curricula	Classes	
Number of classes/workshops per ward	2.125	
Cost per Class (Workshop)	R6,000	10 sessions each class, 40 participants
Cost per ward	R12,750	

These results are then included in the detailed cost profile (under budget category 4.1, ISY SKILLZ).

ISY activities were implemented in the schools. No venue cost was incurred as a result. The venue (school) is another example of a key community resource required for program implementation.

As with community dialogues, school-based dialogues (2 hours, 50 people per dialogue) required the contribution of time by these participants, such as school administrators and teachers. Without the participation of students, the SKILLZ intervention could not be provided (400 hours per SKILLZ workshop; over 800 hours per ward; and over 3,600 hours across the 8 wards).

Category 4.2. Out-of-school Youth (OSY) (Ages 20-24)

The next component of the program was activities targeted to OSY. As with ISY activities, the program included dialogues and the Stepping Stones intervention (and the HIV prevention PP_Prev session included in budget category 6).

The process of organizing the budget information for these dialogues was exactly the same as for the community dialogues (whether a tent was needed for the venue). A dialogue without a tent was R1,750 (refreshments, minor venue fee) and R3,250 if a tent was needed. The table below provides the final cost per dialogue for each implementation district.

From sheet 4.2 OSY – Cost per dialogue for Out-of-School Youth

	Location	Dialogues per ward	Wards Tent	Wards no tent	Cost per dialogue
EThekwini	2	1	3	1	R2,875
Ugu	3	1	1	1	R2,500
UThungulu	4	1	1	1	R2,500

The next intervention targeted to OSY was Stepping Stones (addressing SGBV, Gender Norms). A Stepping Stones workshop involved 10 sessions of 3 hours each.

From Sheet 4.2 OSY – Stepping Stones from the budget

**Conduct 20 x workshops x 10 sessions per group x 40 per group**	**Groups**	**Sessions**	**Units**	**Unit Description**	**Rate**	Total (Rand)
Refreshments @ R15 pp x 20 groups x 10 sessions x 40 per group	20	10	40	participants	15	R120,000
Venue @ R200 p/day x 20 groups x 10 sessions	20	10	1	venue	200	R40,000
**Subtotal**						**R160,000**

With 20 workshops (groups), each with 40 participants, there were on average 2.5 Stepping Stones workshops for each ward, each with a cost (refreshments and venue) of R8,000. These results are then included in the detailed cost profile (budget category 4.2, OSY).

Category 4.3. Adults

The same two interventions targeted to OSY above in Category 4.2, dialogues and Stepping Stones, were also targeted at adults (ages 25+). The cost per dialogue and cost per Stepping Stones workshop were identical (refreshments and venue) to those for OSY. One dialogue per ward was included (as with OSY), but substantially more Stepping Stones workshops were included (7 per ward for adults compared to 2.5 per ward for OSY).

Category 5. Parents

This category included an intervention targeted at parents of the childen participating in the ISY activities (called a parent's workshop). The parents' workshop documentation (see Table 2) states that each workshop involves 5 sessions, 2 or 2.5 hours each. For the CR Program this curriculum was adapted to be covered over 1 session lasting 2 hours.

For this activity, R30 was included per parent for refreshments. With 40 parents per workshop, the cost per workshop was R1,200. A total of 30 workshops, 3.75 per ward on average, were included. These results are then included in the detailed cost profile (budget category 5, parents).

Category 6. HIV Prevention workshops for ISY, OSY, and Adults

The basic HIV prevention intervention included in the program was the 1 session (2 hours) using the PP_Prev Facilitators Guide listed in Table 2. From the SOW, this intervention was targeted 2,495 ISY, 2644 OSY, and 7322 adults. In the budget, R15 per person was included for refeshments. With 40 participants in each prevention session and with R15 per participant for refreshments, the cost per PP_Prev workshop is R600. No venue costs were included. As summarized in the table below, about 8 PP_Prev workshops were organized in each ward for ISY and another 8 for OSY. In addition, about 22 workshops per ward were organized for adults.[1]

ISY	Total learners	2,495
	Learners per session	40
	Total Workshops (1 2-hour session)	62.375
	per ward	7.80
	Cost per Workshop	600
OSY	Total people	2644
	People per session	40
	Total Workshops	66.1
	Workshops per Ward	8.2625
	Cost per workshop	600
Adults	Total people	7322
	People per session	40
	Total Workshops	183.05
	Workshops per ward	22.88125
	Cost per workshop	600

[1] Note that the information presented in the table below on the number of workshops for each sub population is consistent with the amounts included in the budget and the SOW. In the budget, different numbers of workshops are included inside a cell in the workbook, but such numbers were not actually used in the budget calculations.

The information for these PP_Prev workshops are included in the detailed cost profile within the prior budget categories (4.1 for ISY, 4.2 for OSY, and 4.3 for Adults) for easier comparison with the analyses for the other partners where all ISY, OSY, and adult interventions are organized together.

Category 7. HIV demand creation

Budget category 7 is called "HIV demand creation", which involved R6,300 per ward for airtime/data bundles for staff to support linkage to services (e.g. HIV testing) for program participants (e.g., following from the PP_Prev workshop).

Category 8. SGBV Community Mobilization

The first component of this category was additional community dialogues organized to focus on sexual- and gender-based violence (SGBV). Two dialogues per ward were included, and the cost per dialogue were identical (refreshments and venue) to those for OSY, and varied by ward if an additional tent hire cost was required.

In addition, 'small group discussions' were organized for men (ages 20+). Based on information from CCI, these groups used the One Man Can intervention (see Table 2). The information from the budget is provided below.

From sheet 8. SGBV – men's groups from budget

Small group discussion with men (Activity 3.1.1)	Sessions	Groups	Units	Unit Description	Rate	Total
Refreshments @R15.00 pp. x 40 people per small group discussion x 10 groups x 10 sessions	10	10	40	participants	R15	R60,000

The One Man Can intervention (from Table 2) in general involves 5 x 2 hour sessions for 10 hours total. In terms of cost, 10 sessions with R15 per person or 5 sessions with R30 per person is equivalent (R6,000 per group). With 10 workshops, on average there were 1.25 workshops per ward (this is the result of one or two wards that had to have more than one workshop).

Category 9. Monitoring and Evaluation

The remaining budget categories, 9-12, involve general program implementation costs that are not specifically tied to an intervention (listed in Table 2). These budget categories are considered as overall project management costs initially, and simply included as a line item in the detailed cost profile. While minor amounts for subscriptions to an M&E monitoring system were included in the budget, these costs were eventually paid by CCI (and are included later when

considering the additional program costs associated with CCI overall management and supervision).

Category 10. Office operating costs

Category 10 of the budget included other direct costs for DramAidE program management and implementation (see below).

**10. OFFICE OPERATING COSTS**						
	**Units**		**Months**	**LOE**	**RATE**	**TOTAL**
Office Rental	1.00		12	100%	R 11,660.00	R 139,920.00
Building security	1.00		0			R -
Bank charges	1.00		12	100%	R 2,438.00	R 29,256.00
Telecommunications	1.00		12	100%	R 6,000.00	R 72,000.00
Postage & Delivery	1.00		4	100%	R 477.00	R 1,908.00
Printing Reprod Materials	1.00		12	100%	R 2,120.00	R 25,440.00
Insurance - vehicle	2.00		8	100%	R 3,604.00	R 57,664.00
Insurance office furniture and equipment	1.00		12	100%	R 1,060.00	R 12,720.00
Vehicle maintenance and licencing	2.00		8	100%	R 2,703.64	R 43,258.18
Building maintenance & repairs						R -
Computer and Equipment repairs & maintenance	1.00		4	100%	R 530.00	R 2,120.00
Repairs and Maintenance - other						R -
Utilities (1% Operational costs as per constitution)	1.00		1	100%	R 81,797.35	R 81,797.35
Office supplies	1.00		12	80%	R 2,120.00	R 20,352.00
Professional fees - audit	1.00		1	80%	R 50,880.00	R 40,704.00
RCA Audit	1.00		1	100%	R 180,000.00	R 180,000.00

This list of individual line items were then organized into 5 categories: Other equipment (operating costs): Office (audit fees); Office (supplies and other costs); Office rent and related costs; and vehicles (operating costs). The table below shows the link between the individual items above and to which group they are allocated.

From Sheet. 10 Office costs grouped into main categories

HQ Other equipment (operating costs)	Insurance office furniture and equipment
HQ Other equipment (operating costs)	Computer and Equipment repairs & maintenance
HQ Office (audit fees)	Professional fees - audit
HQ Office (audit fees)	RCA audit

HQ Office (supplies and other costs)	Telecommunications
HQ Office (supplies and other costs)	Postage & Delivery
HQ Office (supplies and other costs)	Printing reprod materials
HQ Office (supplies and other costs)	Office supplies
HQ Office rent and related costs	Office rental
HQ Office rent and related costs	Building security
HQ Office rent and related costs	Bank charges
HQ Office rent and related costs	Utilities (1% Operational costs as per constitution)
HQ Vehicles (operating costs)	Insurance - vehicle
HQ Vehicles (operating costs)	Vehicle maintenance and licencing

Aggregating across the four categories, the total for each are provided in the table below.

From Sheet. 10 Office costs grouped into main categories

Category	Total
HQ Other equipment (operating costs)	R14,840
HQ Office (audit fees)	R220,704
HQ Office (supplies and other costs)	R119,700
HQ Office rent and related costs	R250,973
HQ Vehicles (operating costs)	R100,922

These 5 items are then included directly in the detailed cost profile.

Category 11. Equipment

During the program year, the program acquired R434,100 in new equipment, including a vehicle as well as several other durable goods (desks, tables, gazebos). The list is provided below.

From sheet 11. Equipment as budgeted

**11. EQUIPMENT**				
	**Units**	**% to Program**	**RATE**	**TOTAL R**
Containers	3.00	100%	R 36,000.00	R 108,000.00

Computers - desk tops	1.00	100%	R 8,000.00	R 8,000.00
Tables for containers	3.00	100%	R 900.00	R 2,700.00
Chairs for containers (45 x 6)	120.00	100%	R 80.00	R 9,600.00
Fold up table & 4 chairs (HTS mobilization)	8.00	100%	R 3,000.00	R 24,000.00
Gazebos (HTS mobilization)	8.00	100%	R 2,600.00	R 20,800.00
Project Vehicles	1.00	100%	R 261,000.00	R 261,000.00
**TOTAL EQUIPMENT**			**R -**	**R 434,100.00**

In an assessement of program costs for one year, it is common practice to convert this one time payment into an 'annual equivalent cost', which recognizes that these purchases in 2017 will provide program services in future years (assuming the program continued in the future). With a useful life of 5 years and a 5% real discount rate, the annual cost for the vehicle would be R57,413 and R38,077 for the other items.

From sheet 11. Equipment – annualized costs

	total evaluation year	% rate	Project Life (years)	Scrap value	AEC
Equipment (AEC)	173,100	5%	5	0	38,077
Vehicle (AEC)	261,000	5%	5	0	57,413

It would be perhaps easy to suggest that the life of the containers and vehicle could be longer than 5 years, in which case the annual cost were be lower. This annual cost is relatively minor compared to all other costs, so overall results will vary little based on any other reasonable assumption on equipment life and a discount rate.

Category 12. Travel

The last section of the program budget is travel. Information from the budget is provided below.

From sheet 12. Other travel related costs from the budget

**12. TRAVEL AND PER DIEM**					
	**Units**	**Unit description**	**Trip**	**Rate**	**Total**
Travel Re-imbursement (personal vehicles)	12	months	2,000 (kilometers)	R 3.29	78,960
Additional travel for program activities	8	months		R 30,925.63	247,405
**Trips for Program staff to meetings in Pretoria - 4 trips per year for 2 people (2 days per trip)**					

Flights (4 trips for two people) @ R2500 per flight	2	persons	4	R 3,710.00	29,680
Accommodation (3 nights X 4 trips x 2 people) @ R1200per night	6	persons	4	R 901.00	21,624
Per diem (3 nights x 4 trips x 2 people) @ R240 X per day	6	persons	4	R 265.00	6,360
Car hire - 3days x 4 trips @ R 550 X per day	3	days	4	R 530.00	6,360
Parking and Tolls 2 trips per month @ R102 per month	12	months	4	R 159.00	7,632
Fuel for vehicle to project sites	12	months		R 6,360.00	76,320
**Total travel and per Diem**					**R 474,341.00**

The table above is simply copied from the budget. The amounts specified in the first column (e.g., R2,500 per flight) do not correspond to the amounts in the 'Rates' column. The budget for October 2016- September 2017 was the fourth year of the CR program. It is our understanding that the text in first column was copied from prior budget years (but the old amounts were not updated in the text).

There is not additional information to describe the details of the "additional travel for program activities", which is the largest line item in the table above.

This information was organized four main categories as listed below.

From sheet 12. Travel – Main travel cost categories

Travel	HQ travel Pretoria	R71,656
Travel	Reimburse use of personal vehicles	R78,960
Travel	Additional travel program activities	R247,405
Travel	Fuel	R76,320

This information is then included in the detailed cost profile.

Category 13. Annual equivalent cost for other equipment used by program

CCI acquired several computers and two vehicles for use by DramAidE, so these expenditures by CCI are not included in the DramAidE budget. The DramAidE workbook lists these individual items, and the same process was used to estimate an annual equivalent costs for these items as for items in Category 11.

From sheet 13. Other Equipment

Computers AEC	R27,467
Vehicles AEC	R114,827

Summarizing annual program costs – a detailed cost profile (sheet E)

As discussed for each component of the budget (1-13) reviewed above, the number of interventions (e.g., the various dialogues and workshops) and unit costs for each intervention were included in the detailed cost profile (sheet E). This detailed cost profile is also provided as Table 4 below.

It is useful here to review each column in the detailed cost profile. Column A (as noted in Excel) is the category number from the budget. Column B is the basic location where the costs are incurred (1 is headquarters, 2 is EThekwini, 3 is Ugu, and 4 is UThungulu). Whenever it was not obvious where the cost was incurred, the cost was considered a headquarter cost initially. Column C is a simple description of the category.

Column D is just a combination of Column B and C. While the column is redundant in the detailed cost profile, this column is used later for aggregating and summarizing the information in the summary cost profile discussed in the next section (using a pivot table in Excel).

Columns E and F provide additional detail or reminders on the “unit” for the activity. Column G lists the number of each interventions provided per ward, Column H lists the cost per intervention, Column I is the cost per ward, Column J is the number of wards per district (for all partners except Grass Roots Soccer where province is used), and Column K is the total cost per location (2-4 as noted above). For input categories not allocated specifically to wards, a line item aggregate is included in Column K.

Table 4. DramAidE Detailed cost profile for October 2016-September 2017 (Rand)

Budget category	Location	Description	Budget description	Budget detail	Activity Unit	Units per Ward (1 school per ward)	Cost per unit (Rand)	Cost per ward (Rand)	Wards per location (location = province or distict)	Total Rand
1	1	Salary (Management)	1. Salary (Management)	Management						3,621,944
1	1	Salary (Service delivery)	1. Salary (Service delivery)	Service Delivery						1,794,300
3	2	Community Dialogues	3. Community Dialogues	Community Dialogues	Community Dialogue	2	2,875	5,750	4	23,000
3	3	Community Dialogues	3. Community Dialogues	Community Dialogues	Community Dialogue	2	2,500	5000	2	10,000
3	4	Community Dialogues	3. Community Dialogues	Community Dialogues	Community Dialogue	2	2,500	5000	2	10,000
4.1	2	ISY Dialogues	4.1. ISY Dialogues	School Dialogues	School Dialogue	2	750	1500	4	6,000
4.1	3	ISY Dialogues	4.1. ISY Dialogues	School Dialogues	School Dialogue	2	750	1500	2	3,000
4.1	4	ISY Dialogues	4.1. ISY Dialogues	School Dialogues	School Dialogue	2	750	1500	2	3,000
4.1	2	ISY Gender Norms	4.1. ISY Gender Norms	Workshops	SKILLZ 10, 1 hour sessions	2.13	6000	12750	4	51,000
4.1	3	ISY Gender Norms	4.1. ISY Gender Norms	Workshops	SKILLZ 10, 1 hour sessions	2.13	6000	12750	2	25,500
4.1	4	ISY Gender Norms	4.1. ISY Gender Norms	Workshops	SKILLZ 10, 1 hour sessions	2.13	6000	12750	2	25,500
4.1	2	ISY PP Prev	4.1. ISY PP Prev	Sessions	PP Prev 1 2-Hour Sessions	7.80	600	4678	4	18,713
4.1	3	ISY PP Prev	4.1. ISY PP Prev	Sessions	PP Prev 1 2-Hour Sessions	7.80	600	4678	2	9,356
4.1	4	ISY PP Prev	4.1. ISY PP Prev	Sessions	PP Prev 1 2-Hour Sessions	7.80	600	4678	2	9,356
4.2	2	OSY Dialogues	4.2. OSY Dialogues	School Dialogues	School Dialogue	1	2,875	2875	4	11,500
4.2	3	OSY Dialogues	4.2. OSY Dialogues	School Dialogues	School Dialogue	1	2,500	2500	2	5,000
4.2	4	OSY Dialogues	4.2. OSY Dialogues	School Dialogues	School Dialogue	1	2,500	2500	2	5,000

4.2	2	OSY Gender Norms	4.2. OSY Gender Norms	Workshops	Stepping Stones 10 sessions	2.50	8000.00	20000	4	80,000
4.2	3	OSY Gender Norms	4.2. OSY Gender Norms	Workshops	Stepping Stones 10 sessions	2.50	8000.00	20000	2	40,000
4.2	4	OSY Gender Norms	4.2. OSY Gender Norms	Workshops	Stepping Stones 10 sessions	2.50	8000.00	20000	2	40,000
4.2	2	OSY PP Prev	4.2. OSY PP Prev	Sessions	PP Prev 1 2-Hour Sessions	8.26	600	4957.5	4	19,830
4.2	3	OSY PP Prev	4.2. OSY PP Prev	Sessions	PP Prev 1 2-Hour Sessions	8.26	600	4957.5	2	9,915
4.2	4	OSY PP Prev	4.2. OSY PP Prev	Sessions	PP Prev 1 2-Hour Sessions	8.26	600	4957.5	2	9,915
4.3	2	Adults Dialogues	4.3. Adults Dialogues	Adult Dialogue	Adult Dialogue	1	2,875	2875	4	11,500
4.3	3	Adults Dialogues	4.3. Adults Dialogues	Adult Dialogue	Adult Dialogue	1	2,500	2500	2	5,000
4.3	4	Adults Dialogues	4.3. Adults Dialogues	Adult Dialogue	Adult Dialogue	1	2,500	2500	2	5,000
4.3	2	Adults Gender Norms	4.3. Adults Gender Norms	Workhops	Stepping Stones 10 sessions	7.00	8000	56000	4	224,000
4.3	3	Adults Gender Norms	4.3. Adults Gender Norms	Workhops	Stepping Stones 10 sessions	7.00	8000	56000	2	112,000
4.3	4	Adults Gender Norms	4.3. Adults Gender Norms	Workhops	Stepping Stones 10 sessions	7.00	8000	56000	2	112,000
4.3	2	Adults PP Prev	4.3. Adults PP Prev	Sessions	PP Prev 2, 1-Hour Sessions	22.88	600	13728.75	4	54,915
4.3	3	Adults PP Prev	4.3. Adults PP Prev	Sessions	PP Prev 2, 1-Hour Sessions	22.88	600	13728.75	2	27,458
4.3	4	Adults PP Prev	4.3. Adults PP Prev	Sessions	PP Prev 2, 1-Hour Sessions	22.88	600	13728.75	2	27,458
5	2	Adults Parent Workshops	5. Adults Parent Workshops	Workshops	Parent's Guide (1 2 hour session)	3.75	1200	4500	4	18,000
5	3	Adults Parent Workshops	5. Adults Parent Workshops	Workshops	Parent's Guide (1 2 hour session)	3.75	1200	4500	2	9,000

5	4	Adults Parent Workshops	5. Adults Parent Workshops	Workshops	Parent's Guide (1 2 hour session)	3.75	1200	4500	2	9,000
7	2	HIV Prev Demand Creation	7. HIV Prev Demand Creation			1	6300.00	6300	4	25,200
7	3	HIV Prev Demand Creation	7. HIV Prev Demand Creation			1	6300.00	6300	2	12,600
7	4	HIV Prev Demand Creation	7. HIV Prev Demand Creation			1	6300.00	6300	2	12,600
8	2	SGBV Dialogues	8. SGBV Dialogues	Dialogue	SGBV Dialogue	2	2,825	5650	4	22,600
8	3	SGBV Dialogues	8. SGBV Dialogues	Dialogue	SGBV Dialogue	2	2,450	4900	2	9,800
8	4	SGBV Dialogues	8. SGBV Dialogues	Dialogue	SGBV Dialogue	2	2,450	4900	2	9,800
8	2	SGBV Workshops	8. SGBV Workshops	SGBV Men's workshops	Workshop (5 2 hour sessions per workshop)	1.25	6000.00	7500	4	30,000
8	3	SGBV Workshops	8. SGBV Workshops	SGBV Men's workshops	Workshop (5 2 hour sessions per workshop)	1.25	6000.00	7500	2	15,000
8	4	SGBV Workshops	8. SGBV Workshops	SGBV Men's workshops	Workshop (5 2 hour sessions per workshop)	1.25	6000.00	7500	2	15,000
9	1	Office (M&E licenses)	9. Office (M&E licenses)	M&E License fees for accessing system						0
10	1	Office	10. Office	HQ other equipment (operating costs)						14,840
10	1	Office	10. Office	HQ Office (audit fees)						220,704
10	1	Office	10. Office	HQ Office (supplies and other costs)						119,700
10	1	Office	10. Office	HQ Office rent and related costs						250,973

10	1	Office	10. Office	HQ vehicles (operating costs)						100,922
11	1	Equipment (AEC)	11. Equipment (AEC)	HQ New equipment (computers, chairs, etc.) AEC	Computer table, gazbos, etc.					38,078
11	1	Vehicle (AEC)	11. Vehicle (AEC)	New Vehicle AEC	1 Vehicle					57,414
12	1	Travel	12. Travel	HQ travel Pretoria						71,656
12	1	Travel	12. Travel	Reimburse use of personal vehicles						78,960
12	1	Travel	12. Travel	Additional travel program activities						247,405
12	1	Travel	12. Travel	Fuel						76,320
13	1	Prior Equipment (AEC)	13. Prior Equipment (AEC)	Computers purchased previously						27,467
13	1	Prior Vehicle (AEC)	13. Prior Vehicle (AEC)	Vehicles purchased previously						114,827

347

A summary cost profile

With the use of a 'pivot table' in Excel, the above detailed cost profile was converted into a summary cost profile by cost category and location (Table 9 below, sheet C in the DramAidE Workbook). In the table below, salaries for service delivery (e.g., the community mobilizers and facilitators) could have been disaggregaged and allocated to each of the districts (based on the number of wards in each). The process used to estimate costs per ward (at the bottom of Table 5) achieves the same result.

With 8 wards in the program (4 in EThekwini, and 2 each in Ugu and UThungulu), the average program cost per ward (see the bottom of Table 5) is about R1 million per ward in the program. About 15% of these costs are the various venue fees and refreshments for participants; 22% of costs are salaries for the various program facilitators and mobilizers; 45% for other program staff; and the rest (18%) is for various office costs, equipment services, and travel.

Table 5. The Summary Cost Profile

	Headquarters	EThekwini	Ugu	UThungulu	
**Sum of Total Rand**	**Column Labels**				
**Row Labels**	**1**	**2**	**3**	**4**	**Grand Total**
1. Salary (Management)	3,621,944				3,621,944
1. Salary (Service delivery)	1,779,840				1,779,840
10. Office	707,140				707,140
11. Equipment (AEC)	38,078				38,078
11. Vehicle (AEC)	57,414				57,414
12. Travel	474,341				474,341
13. Prior Equipment (AEC)	27,467				27,467
13. Prior Vehicle (AEC)	114,827				114,827
3. Community Dialogues		23,000	10,000	10,000	43,000
4.1. ISY Dialogues		6,000	3,000	3,000	12,000
4.1. ISY Gender Norms		51,000	25,500	25,500	102,000
4.1. ISY PP Prev		9,356	4,678	4,678	18,713
4.2. OSY Dialogues		11,500	5,000	5,000	21,500
4.2. OSY Gender Norms		80,000	40,000	40,000	160,000
4.2. OSY PP Prev		19,830	9,915	9,915	39,660
4.3. Adults Dialogues		11,500	5,000	5,000	21,500
4.3. Adults Gender Norms		224,000	112,000	112,000	448,000
4.3. Adults PP Prev		54,915	27,458	27,458	109,830
5. Adults Parent Workshops		18,000	9,000	9,000	36,000
7. HIV Prev Demand Creation		25,200	12,600	12,600	50,400
8. SGBV Dialogues		22,600	9,800	9,800	42,200
8. SGBV Workshops		30,000	15,000	15,000	60,000
9. Office (M&E licenses)	2,508				2,508
**Grand Total**	**6,823,559**	**586,901**	**288,951**	**288,951**	**7,988,361**

**Wards in programme**	8	4	2	2	
**Cost per ward**	852,945	146,725	144,475	144,475	
**Cost per ward including HQ (shared based on ward numbers)**		**999,670**	**997,420**	**997,420**	

Summarizing Costs and the Intervention Package (from DramAidE as the provider's perspective)

As noted earlier, the CR Program involves a package of interventions. Some of these interventions are conducted over 2 hours per participant, some involve 10 hours per participant, and some 30 hours per participant. Table 10 summarizes total participant hours for each intervention (see also sheet B – Programme in the DramAidE Workbook).

Table 6. The DramAidE intervention package (also sheet B in workbook)

Activiity (number is category in the budget	Activity Detail	Hours of interaction per activity	Participants per Activity	Total participant hours per activity
3. Community Dialogues	Community dialogues with community members and other key local stakeholders.	2	100	200
4.1 ISY Dialogues	Dialogue with educators, parents and learners.	2	50	100
4.1 ISY Gender Norms	Workshop -- SKILLZ (10, 1 hour sessions)	10	40	400
4.1 ISY PP Prev	HIV Prevention Session (PP_Prev facilitators guide)	2	40	80
4.2 OSY Dialogues	Dialogue	2	100	200
4.2 OSY Gender Norms	Stepping Stones workshop	30	40	1200
4.2 OSY PP Prev	HIV Prevention Session (PP_Prev facilitators guide)	2	40	80
4.3 Adults Dialogues	Dialogue (1 day)	2	100	200
4.3 Adults Gender Norms	Stepping Stones (10 1-hour sessions)	30	40	1200
4.4 Adults PP Prev	HIV Prevention Session (PP_Prev facilitators guide)	2	40	80
5. Adults Parent Workshop	Parent's Guide (1 2 hour session)	2	40	80
8. SGBV Dialogues	SGBV Men community dialogues	2	100	200
8. SGBV Workshop	One Man Can (10 hours, 5, 2 hour sessions)	10	40	400

Table 6 shows the total participant hours for one of each intervention in the intervention package. Table 7 (Part A) below also lists the quantities of each intervention provided per ward in the DramAidE program. Since the intensity of each intervention varies (2, 10, 30 hours), and the target audience varies, simply counting the number of intervention workshops is not logical.

As one possibility, we used Table 6 and Table 7 (Part A) to estimate the total participant hours (or program hours) for each program component in Table 7 (Part B) – also see sheet D – cost outputs per ward. For each ward, we estimate 17,500 participant hours per ward for the DramAidE program. If the various dialogues are not considered actual 'interventions', but more as necessary activities needed to be able to implement the other interventions, then intervention hours fall to about 16,000 hours. Not surprisingly, for a community-based program, substantial participation by community members is required.

At the bottom of Table 7 (Part C. Final Summary), with a cost per ward of approximately R1 million and 16,165 participant hours, the cost per participant hour is R62.

Table 7. Summary Results for DramAide: Intervention package quantities, participant hours, and cost per ward.

**PART A. Intervention**	**Number per ward**
Community Dialogues	2.0
ISY Dialogues	2.0
ISY Gender Norms	2.1
ISY PP Prev	7.8
OSY Dialogues	1.0
OSY Gender Norms	2.5
OSY PP Prev	8.3
Adults Dialogues	1.0
Adults Gender Norms	7.0
Adults PP Prev	22.9
Adults Parent Workshop	3.8
SGBV Dialogues	2.0
SGBV Workshop	1.3
**PART B. Intervention**	**Total participant hours**
Community Dialogues	400
ISY Dialogues	200
ISY Gender Norms	850
ISY PP Prev	624
OSY Dialogues	200
OSY Gender Norms	3,000
OSY PP Prev	661
Adults Dialogues	200
Adults Gender Norms	8,400
Adults PP Prev	1,831
Adults Parent Workshop	300
SGBV Dialogues	400
SGBV Workshop	500
**Part C. Final Summary**	
Total hours all programme activities	17,565
Total intervention hours (excludes dialogues)	16,165

Total cost per ward	R 999,670
Total cost per participant intervention hour	R 62

Last Step – summarizing costs from USAID's perspective

The total cost per ward of approximately R1,000,000 is the estimated cost from the DramAidE perspective, which as noted earlier excludes a 'cost' for inputs contributed to the program by others (the local community/schools for venues, community members time for dialogues, and the basic time provided to the program by participant).

In addition, DramAidE would not have been able to implement the CR Programme without the contributions of CCI as the prime recipient of the overall funding from USAID. During FY17, the CCI component of the CR Programme was R7,284,981. Assuming that each partner benefited equally from CCI activities, the additional CCI cost per partner was R 1,214,163.

For DramAidE, with 8 wards in the program, this impies an additional cost per ward of R151,770.[2] With this additional CCI contribution allocated to each partner, the cost per participant hour for DramAidE would increase to R71 (an additional cost of R9 per participant hour).

[2] Note that any annual equivalent cost for equipment purchased in prior program years, or used by CCI but not purchased by the program, is excluded from this simple analysis.
