# Supplementary material for: Understanding the costs and the cost structure of a community-based HIV and gender-based violence (GBV) prevention program: the Woza Asibonisane Community Responses Program in South Africa
Source: BMC Health Serv Res. 2020 Jun 10;20:526. doi: 10.1186/s12913-020-05385-1 (PMC7288692; doi:10.1186/s12913-020-05385-1)
Supplement: Supplementary file 3 — Additional file 3. Summary cost profiles of implementing NGOs. [file 12913_2020_5385_MOESM3_ESM.pdf]

Summary cost profiles of NGOs and cost per participant hour by activity disaggregated and by cost category

Table 1. ISO Summary Cost Profile

**ISO**			
**Headquarters**	**Rand 2017**	**Rand 2017**	**Share of total participant hours**
Salary (Management)	942 934	942 934	
Salary (Service delivery)	1 476 092	1 476 092	
Office	363 042	581 560	
Office (M&E licenses)	2 645		
Equipment (AEC)	127 043		
Vehicle (AEC)	0		
Travel	39 480		
Fuel	49 350		
Prior Equipment (AEC)	0		
Prior Vehicle (AEC)	0		
HIV Prev Demand Creation**	0		
**Direct***			
Community Dialogues	43 216	43 216	0.042
ISY Dialogues	15 450	15 450	0.016
ISY Gender Norms	2 060	2 060	0.141
ISY PP Prev	74 933	74 933	0.086
OSY Dialogues	14 008	14 008	0.021
OSY Gender Norms	14 008	14 008	0.120
OSY PP Prev	87 648	87 648	0.100
Adults Dialogues	14 008	14 008	0.021
Adults Gender Norms	25 544	25 544	0.110
Adults PP Prev	193 542	193 542	0.222
Adults Parent Workshops	25 544	25 544	0.022
GBV Dialogues	49 440	49 440	0.085
GBV Workshops	4 841	4 841	0.013
Total	3 564 827	3 564 827	
* Direct costs were refreshments for participants and minor venue costs for some non-school locations			
** Cell phone top up time for staff to link PP Prev participants to HIV testing locations.			

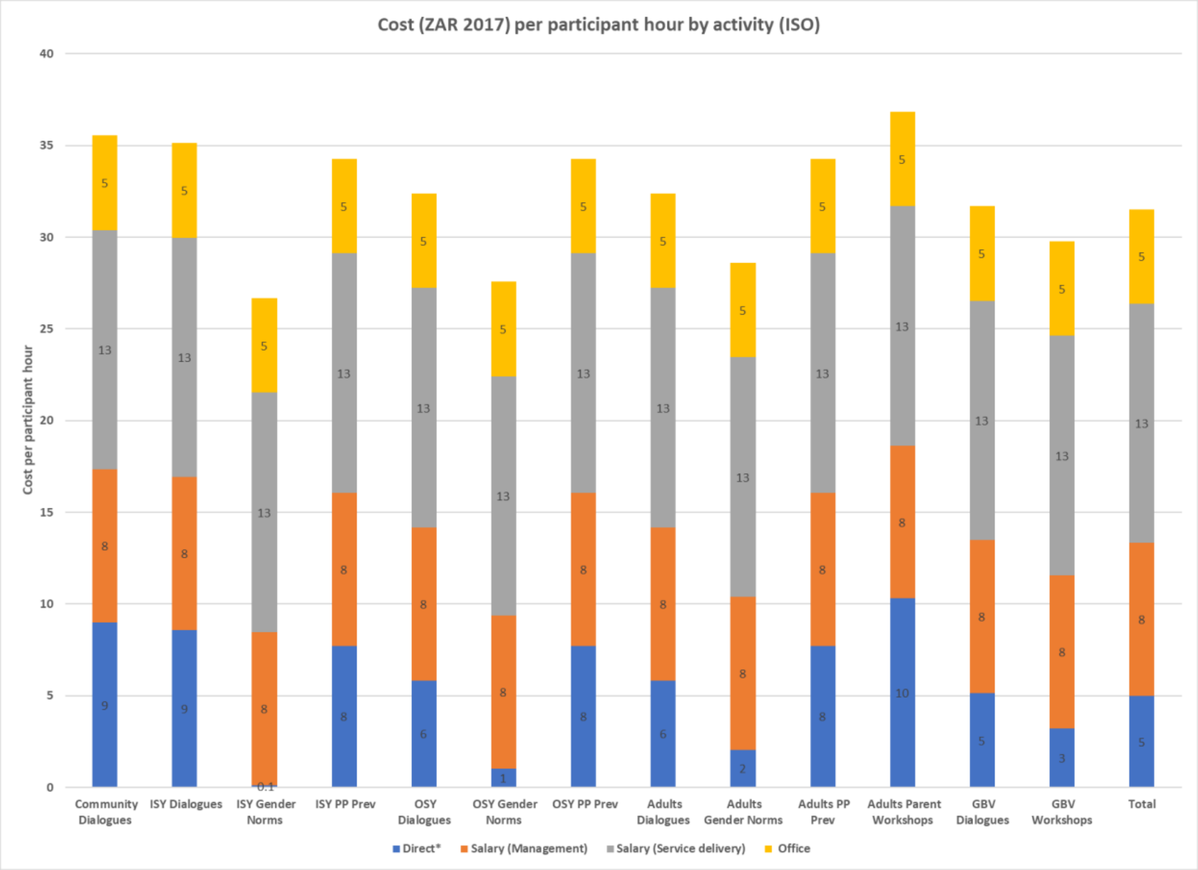

Figure 1: Cost per participant hour by activity disaggregated by cost category (ISO)

Table 2. TVT Summary Cost Profile

**TVT**			
**Headquarters**	**Rand 2017**	**Rand 2017**	**Share of total participant hours**
Salary (Management)	1 348 605	1 348 605	
Salary (Service delivery)	2 186 851	2 186 851	
Office	611 603	1 020 238	
Office (M&E licenses)	2 664		
Equipment (AEC)	265 954		
Vehicle (AEC)	0		
Travel	78 960		
Fuel	61 056		
Prior Equipment (AEC)	0		
Prior Vehicle (AEC)	0		
HIV Prev Demand Creation**	0		
**Direct***			

Community Dialogues	51 800	51 800	0.040
ISY Dialogues	14 310	14 310	0.018
ISY Gender Norms	1 177	1 177	0.129
ISY PP Prev	22 292	22 292	0.028
OSY Dialogues	17 900	17 900	0.020
OSY Gender Norms	17 120	17 120	0.159
OSY PP Prev	37 827	37 827	0.037
Adults Dialogues	17 900	17 900	0.020
Adults Gender Norms	328 000	328 000	0.397
Adults PP Prev	90 079	90 079	0.085
Adults Parent Workshops	7 632	7 632	0.010
GBV Dialogues	25 900	25 900	0.020
GBV Workshops	6 461	6 461	0.039
Total	5 194 091	5 194 091	
* Direct costs were refreshments for participants and minor venue costs for some non-school locations			
** Cell phone top up time for staff to link PP Prev participants to HIV testing locations.			

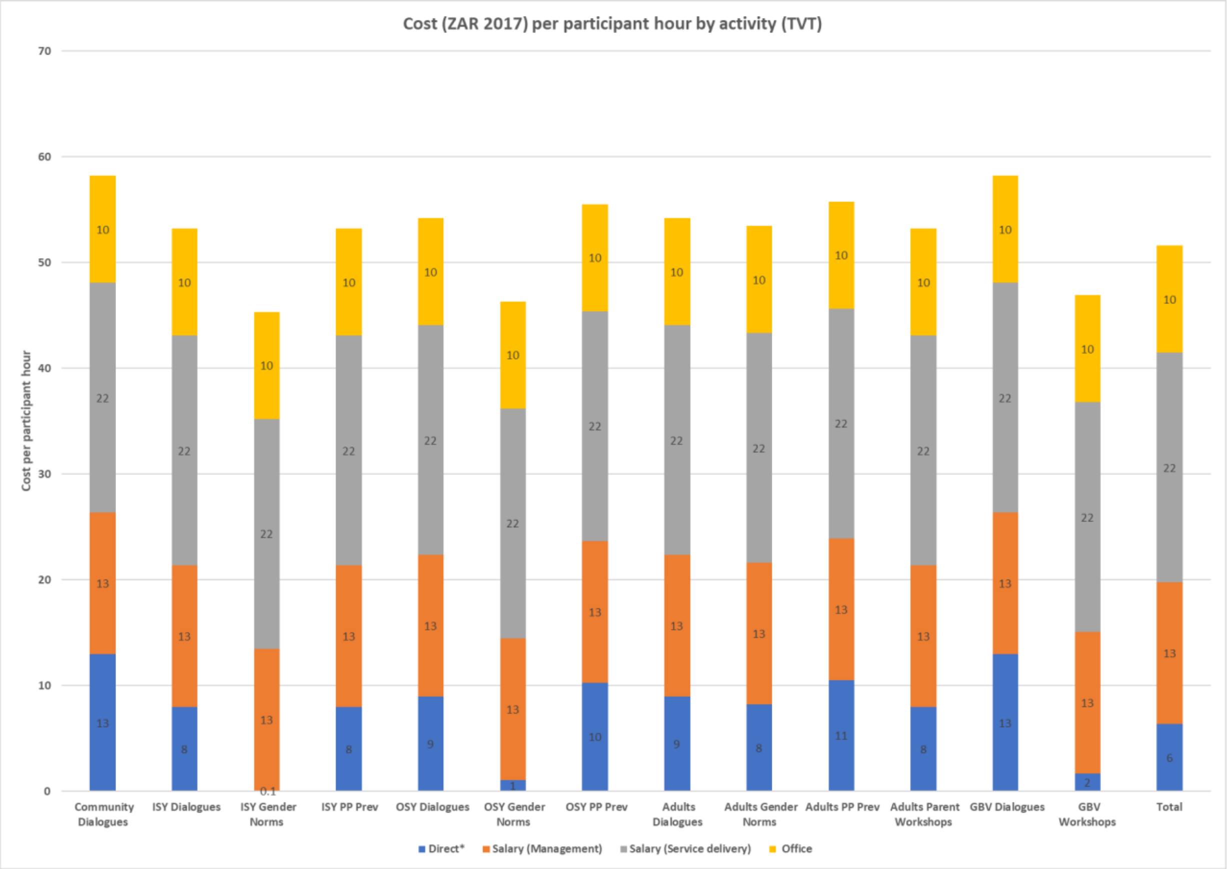

Figure 2: Cost per participant hour by activity disaggregated by cost category (TVT)

Table 3. PSASA Summary Cost Profile

PSASA			
**Headquarters**	**Rand 2017**	**Rand 2017**	**Share of total participant hours**
Salary (Management)	2 208 377	2 208 377	
Salary (Service delivery)	2 469 593	2 469 593	
Office	639 426	1 214 512	
Office (M&E licenses)	2 340		
Equipment (AEC)	21 064		
Vehicle (AEC)	106 474		
Travel	384 211		
Fuel	60 997		
Prior Equipment (AEC)	0		
Prior Vehicle (AEC)	0		
HIV Prev Demand Creation**	0		
**Direct***			
Community Dialogues	20 400	20 400	0.014
ISY Dialogues	21 000	21 000	0.016
ISY Gender Norms	168 000	168 000	0.197
ISY PP Prev	86 010	86 010	0.101
OSY Dialogues	10 200	10 200	0.007
OSY Gender Norms	208 000	208 000	0.183
OSY PP Prev	87 470	87 470	0.098
Adults Dialogues	10 200	10 200	0.007
Adults Gender Norms	216 000	216 000	0.190
Adults PP Prev	88 270	88 270	0.132
Adults Parent Workshops	16 800	16 800	0.010
GBV Dialogues	40 800	40 800	0.028
GBV Workshops	12 600	12 600	0.016
Total	6 878 231	6 878 231	
* Direct costs were refreshments for participants and minor venue costs for some non-school locations			
** Cell phone top up time for staff to link PP Prev participants to HIV testing locations.			

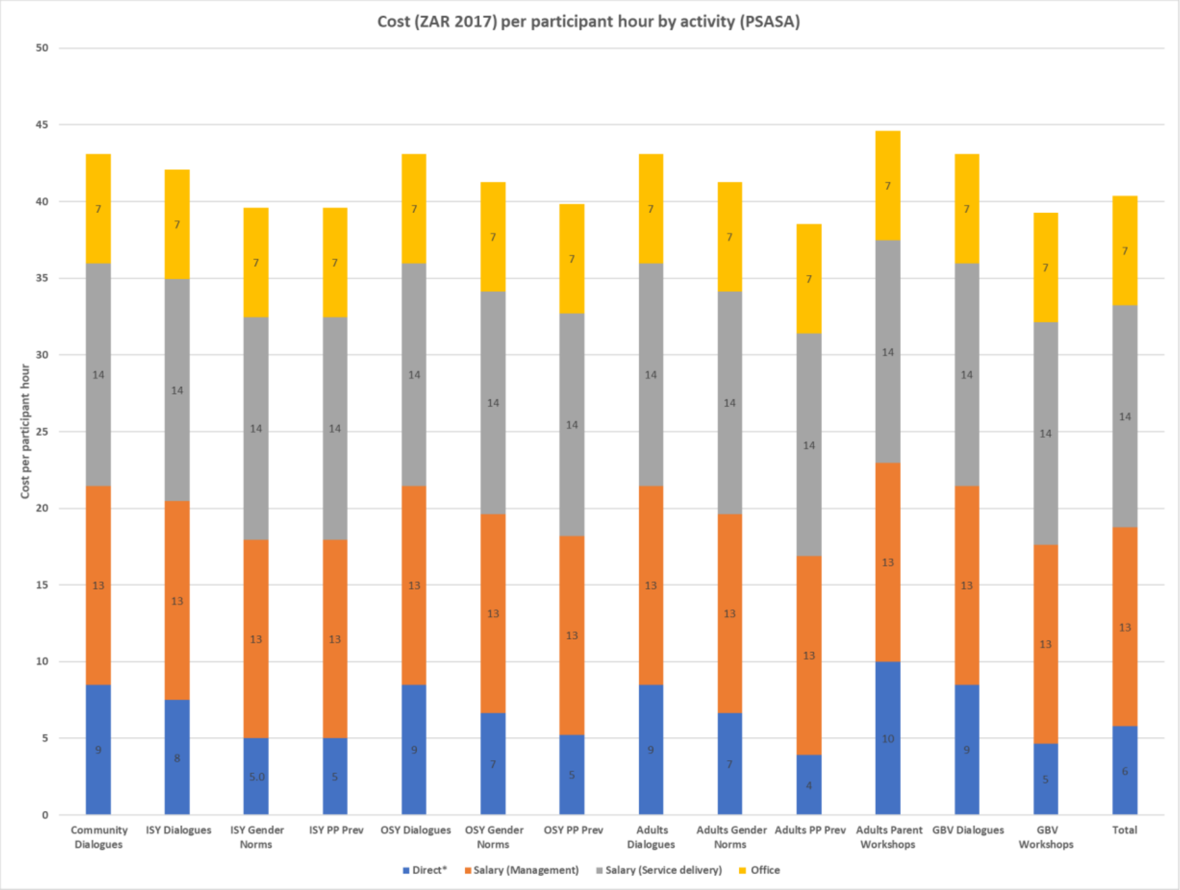

Figure 3: Cost per participant hour by activity disaggregated by cost category (PSASA)

Table 4. HDF Summary Cost Profile

HDF			
**Headquarters**	**Rand 2017**	**Rand 2017**	**Share of total participant hours**
Salary (Management)	1 129 282	1 129 282	
Salary (Service delivery)	1 781 054	1 781 054	
Office	497 786	791 490	
Office (M&E licenses)	2 645		
Equipment (AEC)	31 357		
Vehicle (AEC)	78 971		
Travel	0		
Fuel	180 731		
Prior Equipment (AEC)	0		
Prior Vehicle (AEC)	0		
HIV Prev Demand Creation**	0		
**Direct***			
Community Dialogues	31 518	31 518	0.036
ISY Dialogues	7 725	7 725	0.010
ISY Gender Norms	30 900	30 900	0.060
ISY PP Prev	40 000	40 000	0.040
OSY Dialogues	15 759	15 759	0.018
OSY Gender Norms	74 160	74 160	0.121
OSY PP Prev	67 080	67 080	0.060
Adults Dialogues	15 759	15 759	0.018
Adults Gender Norms	228 866	228 866	0.374
Adults PP Prev	192 723	192 723	0.172
Adults Parent Workshops	17 600	17 600	0.018
GBV Dialogues	31 286	31 286	0.072
GBV Workshops	26 780	26 780	0.024
Total	4 481 982	4 481 982	
* Direct costs were refreshments for participants and minor venue costs for some non-school locations			
** Cell phone top up time for staff to link PP Prev participants to HIV testing locations.			

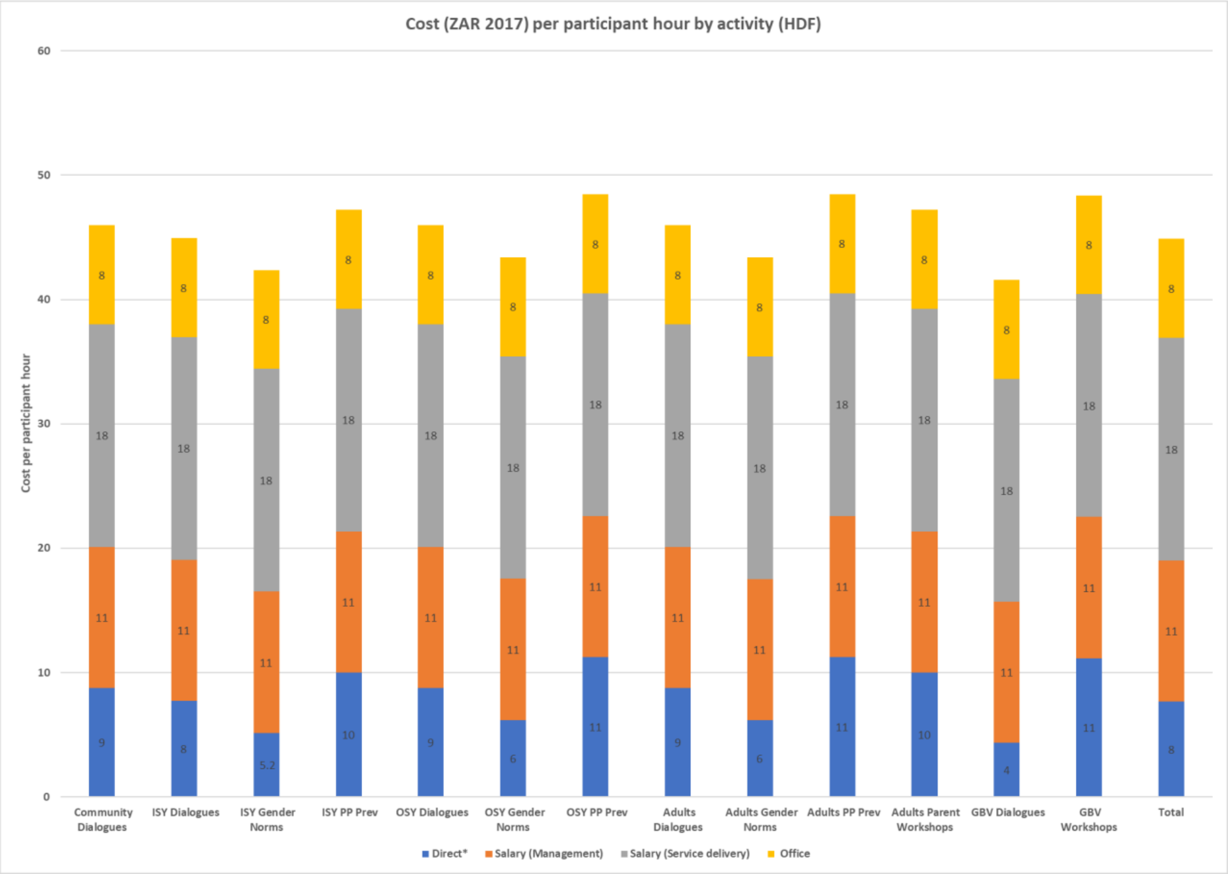

Figure 4: Cost per participant hour by activity disaggregated by cost category (HDF)

Table 5. GRS Summary Cost Profile

**GRS**			
**Headquarters**	**Rand 2017**	**Rand 2017**	**Share of total participant hours**
Salary (Management)	3 193 165	3 193 165	
Salary (Service delivery)	3 023 194	3 023 194	
Office	1 173 260	1 516 975	
Office (M&E licenses)	0		
Equipment (AEC)	60 658		
Vehicle (AEC)	83 591		
Travel	122 834		
Fuel	76 632		
Prior Equipment (AEC)	0		
Prior Vehicle (AEC)	0		
HIV Prev Demand Creation**	0		
**Direct***			
Community Dialogues	28 840	28 840	0.017

ISY Dialogues	12 360	12 360	0.009
ISY Gender Norms	173 040	173 040	0.060
ISY PP Prev	114 783	114 783	0.059
OSY Dialogues	14 420	14 420	0.009
OSY Gender Norms	173 040	173 040	0.179
OSY PP Prev	155 200	155 200	0.080
Adults Dialogues	14 420	14 420	0.009
Adults Gender Norms	395 829	395 829	0.403
Adults PP Prev	176 336	176 336	0.091
Adults Parent Workshops	35 226	35 226	0.024
GBV Dialogues	57 680	57 680	0.034
GBV Workshops	52 530	52 530	0.027
Total	9 137 038	9 137 038	
* Direct costs were refreshments for participants and minor venue costs for some non-school locations			
** Cell phone top up time for staff to link PP Prev participants to HIV testing locations.			

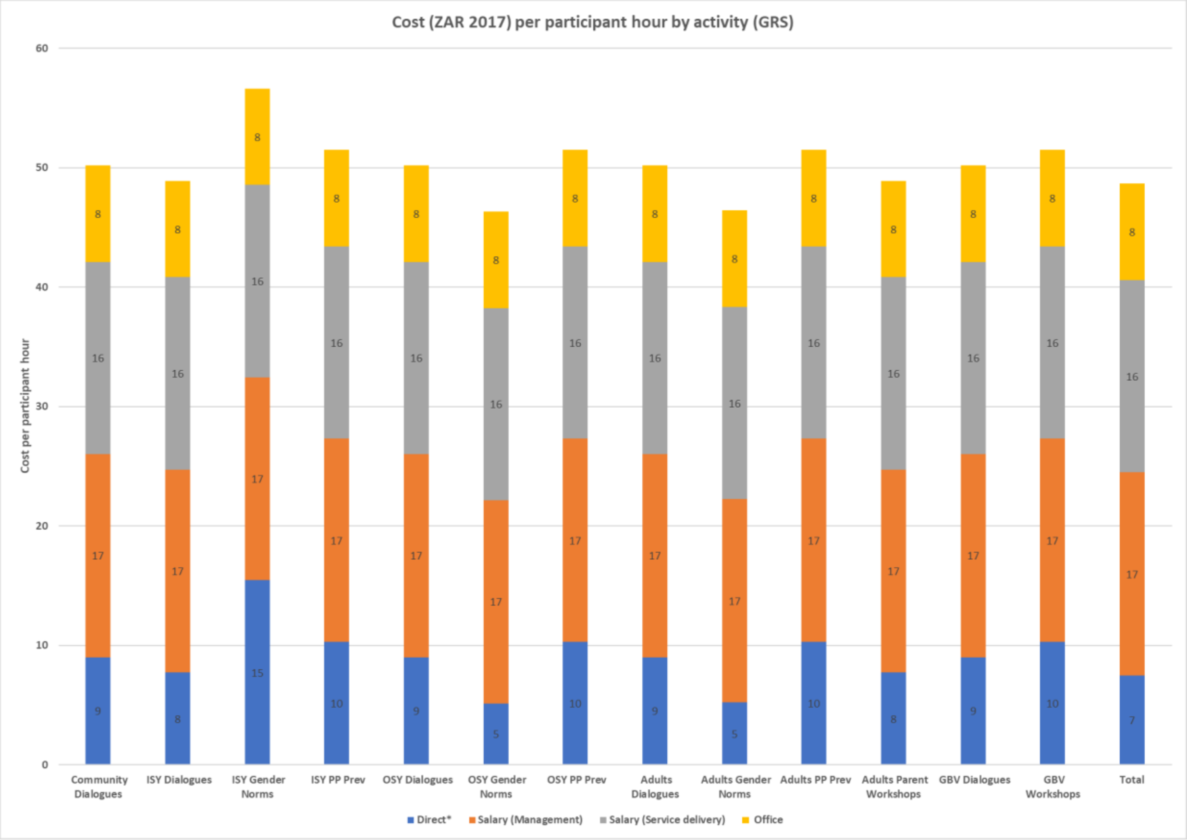

Figure 5: Cost per participant hour by activity disaggregated by cost category (GRS)
